# The impact of pooling on the observed microbiome profile of preweaned piglet feces

**DOI:** 10.1093/femsec/fiaf058

**Published:** 2025-06-06

**Authors:** Tara N Gaire, Jared Young, Thomas Wehri, Mark Schwartz, Randall Singer, Maria Pieters, Noelle R Noyes

**Affiliations:** Department of Veterinary Population Medicine, College of Veterinary Medicine, University of Minnesota, Saint Paul, MN, 55108, United States; Department of Veterinary Population Medicine, College of Veterinary Medicine, University of Minnesota, Saint Paul, MN, 55108, United States; Department of Veterinary Population Medicine, College of Veterinary Medicine, University of Minnesota, Saint Paul, MN, 55108, United States; Department of Veterinary Population Medicine, College of Veterinary Medicine, University of Minnesota, Saint Paul, MN, 55108, United States; Schwartz Farms Inc, Sleepy Eye, MN, 56085, United States; Swine Disease Eradication Center, College of Veterinary Medicine, University of Minnesota, Saint Paul, MN, 55108, United States; Department of Veterinary and Biomedical Sciences, College of Veterinary Medicine, University of Minnesota, Saint Paul, MN, 55108, United States; Department of Veterinary Population Medicine, College of Veterinary Medicine, University of Minnesota, Saint Paul, MN, 55108, United States; Swine Disease Eradication Center, College of Veterinary Medicine, University of Minnesota, Saint Paul, MN, 55108, United States; Department of Veterinary Population Medicine, Veterinary Diagnostic Laboratory, College of Veterinary Medicine, University of Minnesota, Saint Paul, MN, 55108, United States; Department of Veterinary Population Medicine, College of Veterinary Medicine, University of Minnesota, Saint Paul, MN, 55108, United States

**Keywords:** 16S rRNA gene sequencing, composite pen floor, fecal microbiome, pooling, swine

## Abstract

Pooling individual samples could be an efficient approach for large-scale population-based microbiome studies. However, it is unknown whether pooled samples accurately reflect the microbiome composition and diversity obtained from individual samples. This study investigated the impact of various pooling methods on the observed fecal microbiome of preweaned piglets. Individual fecal samples were collected from 10 litters of day-old piglets (*N* = 137) and 10 litters of 20-day-old piglets (*N* = 121), as well as pen-floor samples from the same litters. The individually collected samples were processed individually and also used to create pools of both raw feces and extracted DNA. Individual samples, raw feces pools, DNA pools, and pen-floor samples were subjected to 16S rRNA gene sequencing. The microbial profile in pen-floor samples from litters of preweaned piglets was very different from individual piglet samples within the pen; thus, they may not be suitable for litter-level piglet microbiome studies. However, overall microbial diversity and composition from DNA and feces pools were comparable to individual samples, despite potentially underestimating some low-abundance or low-prevalence taxa. These results suggest that pooling can be used as an efficient and cost-effective approach to characterize litter-level microbial profiles for current and future population-level microbiome research in preweaned piglet populations.

## Teaser

Pooling raw feces or DNA from individual samples can be considered for estimating diversity, abundance, and composition in litter-level microbiome studies of preweaned piglet populations.

## Introduction

The fecal microbiome exhibits substantial interindividual variability (Salonen et al. 2014). This variability manifests as differences in overall microbial composition, diversity, and relative abundance of individual taxa across individuals. Consequently, microbiome studies typically involve collecting, processing, and sequencing samples at the individual host level (Laukens et al. 2016, Gilbert et al. 2018). In livestock microbiome studies, however, interventions are often applied at the group level (e.g. herd, flock, or pen), and the unit of interest is typically the group rather than the individual. Under such circumstances, pooling samples from multiple individuals could be a cost-effective method to study the microbiome at the group level. However, the impact of pooling on microbiome metrics such as diversity and composition has not been fully explored.

Pooled testing offers an efficient method for large-scale studies by combining individual samples into a group or pool for analysis, rather than testing each sample separately (Furstenau et al. 2020, Regen et al. 2020, Cleary et al. 2021). In veterinary medicine, pooled samples, often referred to interchangeably as composite samples, have traditionally been used for pathogen detection and infection status assessment in herds, populations, or animal products (Jordan 2005, Singer et al. 2006, Reyher and Dohoo 2011, Lombard et al. 2012, Ronco et al. 2018). For many sample types, specimens collected from individual animals are combined to create a pooled sample; for example, blood or serum samples collected from individual animals are mixed together to create a pool. Fecal samples, however, are often collected from the housing environment of a group of animals to create a composite or pooled sample, rather than being collected directly from the rectum of individual animals. In microbiome studies, the impact of pooling may differ from its use in traditional applications, such as diagnostic testing, where pooling is primarily used to detect a specific pathogen. In microbiome research, the focus is not on a single pathogen of interest, but rather on understanding the composition and diversity of microbial communities within a host population or environment. Thus, the success of pooling in microbiome research relies on its ability to represent the diverse microbial populations observed in individual samples within the pool. Despite the long-standing use of pooled testing in diverse settings, its application in microbiome studies is very limited (Munk et al. 2017, Ray et al. 2019). For example, Ray et al. (2019) demonstrated the potential utility of pooled DNA samples to estimate community-level diversity in population-level human microbiome studies. Similarly, Munk et al. (2017) found that pig pen-floor samples can represent the fecal/rectal microbial profile obtained from individual pig samples. These findings indicate that pooled samples could be considered for livestock microbiome research, but further investigation is needed to understand the feasibility and limitations of comparing microbiome profiles obtained from pooled versus individual samples.

Pooling can occur at multiple points along the sample collection and processing workflows. At sample collection, the use of composite pen-floor fecal samples for microbiome analysis has been described in finisher pigs (Munk et al. 2017). Pen-floor sampling provides a convenient sampling protocol with minimal animal handling, which minimizes animal stress and labor requirements. However, pen-floor composite samples may not accurately reflect the fecal microbiome of preweaned piglets because they are cohoused with their sow, and the sow’s feces likely represents a large proportion of the fecal material on the pen floor. The utility of pen-floor composite fecal samples for preweaned piglets has not yet been described. Pooling can also be performed on samples collected from individuals, for example by pooling raw fecal materials prior to genomic DNA extraction, or by pooling DNA extracted from individual samples. The use of these pooling strategies for preweaning piglet microbiome research has not yet been described in the scientific literature. Understanding the impact of various pooling strategies is crucial for the practical application of the pooling approach in field-based microbiome studies and may aid in decision-making regarding whether pooling can be used as an alternative to individual samples for large field-based swine fecal microbiome studies.

Therefore, the aim of this study was to evaluate the impact of various pooling methods on the observed fecal microbiome of preweaned piglets. We hypothesized that the microbial composition and diversity of pooled samples would be statistically and biologically similar to those of individual samples, given the shared pen environment among piglets within the same litter. To investigate this, we collected individual fecal and pen-floor composite fecal samples from both day-old and 20-day-old litters of piglets from a single commercial swine farm. Individually collected samples were pooled both using raw feces and extracted DNA. Individual samples, fecal pools, DNA pools, and composite pen-floor libraries were subjected to 16S rRNA gene sequencing and the microbial profiles were compared across workflows.

## Methods

### Study population

This study was approved by the Institutional Animal Care and Use Committee (IACUC) (Protocol ID: 2010–38537A) of the University of Minnesota. In this cross-sectional observational study, 20 litters of piglets from a single commercial sow facility were enrolled into the study on a single sampling day. Litters were selected based on convenience, and were housed in two rooms. None of the enrolled sows had received any antimicrobial drugs during lactation and for at least 1 week prior to farrowing, and enrolled piglets had not received any antimicrobial drugs during their lifespan. Throughout lactation, sows were fed a corn–soybean meal-based diet supplemented with 15% corn dried distillers grains with solubles (Table S1). The diet was formulated to meet or exceed the nutrient requirements established by the National Research Council (NRC 2012). Piglets from all litters suckled *ad libitum* during lactation and were provided creep feed starting at 17–18 days of age (Table S2).

On the day of sampling, 10 of the enrolled litters contained 1-day-old piglets in one room, and the other 10 litters contained 20-day-old piglets (i.e. 1 day prior to weaning age) in another room. The mean litter size of enrolled litters at the time of sampling was 13 piglets (median and mode both 14, range 8–16 piglets). The enrolled piglets were born to multiparous sows and were not cross-fostered. They were processed alongside other piglets within the room at 2–5 days postbirth, which included an iron injection, tail docking for all piglets, and castration for barrows, following farm protocols. The piglets included in the study were healthy at the time of sampling, with no signs of diarrhea or respiratory disease observed in any of the enrolled piglets.

### Sample collection

#### Individual *per-rectum* fecal swabs

All piglets from all litters were individually sampled by trained personnel. Samples were collected by inserting a sterile cotton-tipped swab (Hardy Diagnostics, catalog #HD258062WC) ~2–4 cm into the rectum and gently rotating the swab. Gloves were changed between each rectal swab sample, and sampled piglets were marked on their dorsum with chalk. Immediately after collection, each swab was placed into a sterile Whirl-Pak bag and labeled with litter identification number, room number, and date of collection. A total of 258 individual rectal swabs were obtained from 258 individual piglets (day-old piglets *N* = 137, 20-day old piglets *N* = 121).

#### Composite pen-floor samples

Composite pen-floor swab samples of fecal matter on the pen floor were collected from each litter using sterile cotton-tipped swabs. At the same time at which individual fecal swabs were collected, and for each enrolled litter, three swabs were used to swab fecal matter from three areas of the pen floor—one near the sow feeder (front area) and the two corners at the rear of the pen. During swabbing, the swab was rotated multiple times at each sampling area while avoiding contact with the sow, piglets, or pen railings. The pen-floor fecal material varied in texture, ranging from soft/loose to, in some cases, a mixture with fluid. A total of 60 pen-floor samples were collected (20 litters, 3 swabs per litter). Immediately after swabbing, each swab was placed in a sterile Whirl-Pak bag and the bag was labeled with litter identification number, room number, and date of collection.

#### Sampling blanks

During piglet sampling, four sampling controls were collected by gently waving sterile swabs in the sampling room, in the vicinity of the sampled litters. The swabs were then placed into a sterile Whirl-Pak bag and labeled as negative control sampling blanks. These sampling blanks were used to identify any potential sources of contamination (see details in decontam and source tracker models).

All samples were transported on ice to the Food Centric Corridor at the University of Minnesota and stored within 4 h of sampling at −80°C until further processing.

### Sample processing and DNA extraction workflow

**Figure 1.**
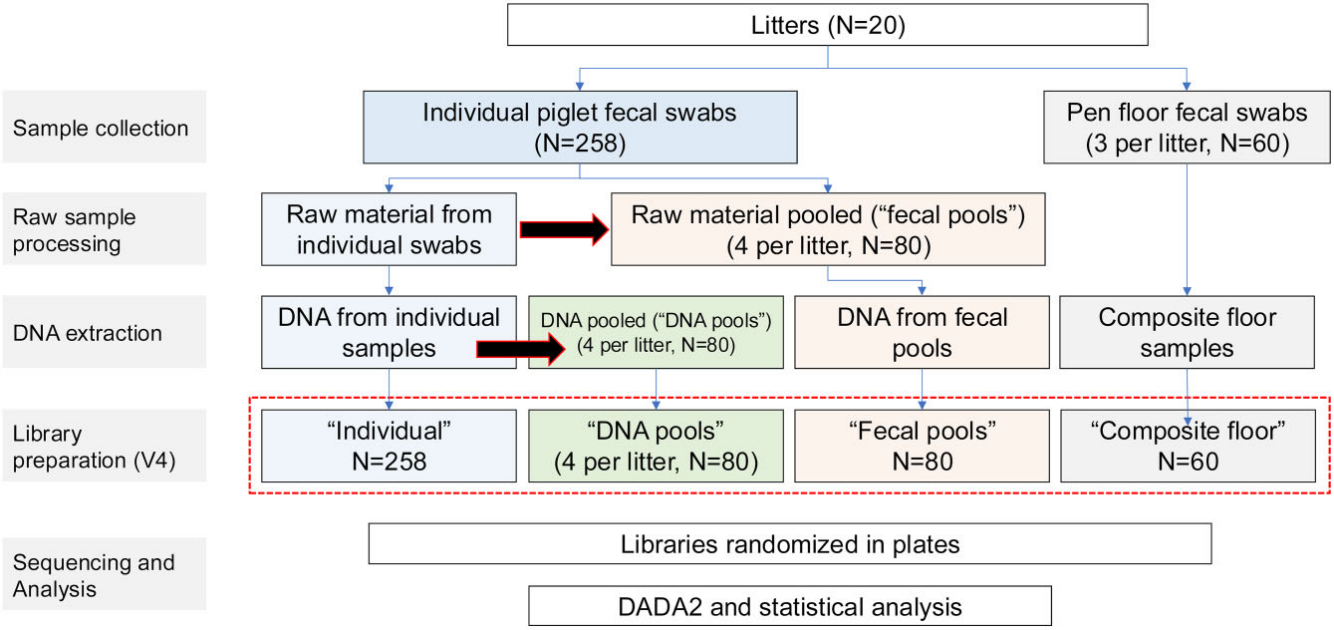
Pooling workflow.

Our objective was to evaluate the effect of different pooling methods on the observed fecal microbiome of litters of piglets. Thus, we compared the fecal microbiome obtained from samples processed using both individual and pooled workflows using four different methods (i.e. pooled workflows; Fig. 1, Fig. S1): (i) individual samples were processed for DNA extraction (“individual,” *N* = 258), (ii) individual samples were pooled (4 pools/litter) prior to DNA extraction (“fecal pools,” *N* = 80), (iii) DNA was extracted from individual samples and then pooled for sequencing (4 pools/litter), (“DNA pools,” *N* = 80); and (iv) composite pen-floor samples were processed for DNA extraction (“composite floor,” 3/litter, *N* = 60). An equal-volume pooling approach (as opposed to an equal-concentration approach) was chosen to create DNA pools from individually extracted DNA, irrespective of variations in DNA concentration. This approach was selected to simplify the pooling workflow and maintain consistency across all workflows, as this allows input volumes to be adjusted without the need to measure DNA concentration for each sample. In contrast, an equal-concentration approach is time-consuming and has potential discrepancies in volume for each sample to adjust concentration. In our other pooled workflows (fecal pools and composite pen-floor samples), this equal-concentration DNA pooling was not relevant, as equal volumes of raw fecal material from individual samples were combined to create the fecal pools prior to extraction, while composite pen-floor samples were processed individually for DNA extraction (details below). Thus, to avoid potential discrepancies for each workflow, the equal-volume pooling approach aligns with our aim to maintain a consistent method across all workflows and simplify the overall pooling process, with the ultimate goal of supporting epidemiological studies involving large sample numbers.

“Individual” samples represent rectal swabs collected from individual piglets, and were processed as individual samples throughout the entire workflow (*N* = 258 from 20 litters). These swabs were processed individually for genomic DNA extraction using the DNeasy PowerSoil Pro Kit (Qiagen, catalog number 47016, Hilden, Germany), with slight modifications from the manufacturer’s instructions; specifically we increased the lysis buffer volume (i.e. CD1 buffer) to 1000 µl instead of 800 µl. This adjustment was made to accommodate a sufficient volume of raw feces, ensuring that the raw feces from each individual sample could also be used to create a fecal pool (see details below). Briefly, each rectal swab was carefully removed from the Whirlpool bags, and the swab tips were detached and placed into a PowerBead tube containing 1000 µl of CD1 lysis buffer. Subsequently, the PowerBead tubes were vortexed using the Mini Beadbeater^TM^ at 2200 r/m for 20 s per cycle, for a total of three cycles, with a 30-s pause between each cycle. After centrifugation at 16 000 × *g* for 1 min and 30 s, 600 µl of the fecal supernatant was transferred to the second position of the rotor adapter. The remaining supernatant was used to create “fecal pools” (see below). DNA extractions were performed on two QIAcube machines (Qiagen, catalog number 9002864). All samples were processed with Inhibitor Removal Technology to eliminate inhibitors. Extracted DNA was eluted in a 50 µl elution buffer, and tubes containing DNA were labeled and stored at −20°C until 16S rRNA library preparation and sequencing.

“Fecal pools” were created during the processing of individual samples. Thus, the fecal pool and individual samples were processed simultaneously, avoiding additional freezing and thawing of the samples. Once all individual piglet samples within a given litter were processed as described above, litter-level fecal pools were formed by combining equal volumes of fecal supernatant from each piglet in that litter. Piglets included in each pool were not randomly selected but rather chosen haphazardly from the list of piglets in that litter. For each litter, four fecal pools were created, regardless of litter size. Each pool was adjusted to a final volume of 600 µl, consistent with the volume specified by the Qiacube protocol. For example, in a litter of 8 piglets, four fecal pools were formed, each consisting of 300 µl of supernatant from each of 2 piglets. For a litter of 14 piglets, the first three pools contained supernatant from 4 piglets each (150 µl each), and the fourth pool contained supernatant from 2 piglets (300 µl each). These fecal pools, each containing 600 µl, were then transferred to the second position of each rotor adapter, and the subsequent extraction steps were performed using the Qiacube, following the same procedures as for individual samples.

“DNA pools” were created once DNA was extracted from individual samples. DNA pools were created using the same procedure (i.e. equal volume) as for fecal pools, including using the same piglets within each pool, rather than selecting them randomly. For instance, if piglet IDs 1, 2, 3, and 4 from litter 1 were used to create one fecal pool, then DNA from the same individual piglets (i.e. 1–4) was pooled to make one corresponding DNA pool.

“Composite floor” swabs were processed similarly to individual samples by adding 1000 µl of CD1 solution, as described above, thus generating three observations per litter.

During the sample processing for DNA extraction, extraction blanks (*N* = 7) were created by adding 1000 µl of CD1 solution (DNeasy PowerSoil Pro Kit, Qiagen, catalog number 47016) to a PowerBead tube, without any sample. Two ZymoBIOMICS® Microbial Community Standards (Log Distribution, catalog number D6310, Zymo Research Corp., Irvine, USA) were included as positive controls for DNA extraction and sequencing. These mock communities were processed using the DNeasy PowerSoil Pro Kit protocol, with the exception that a 200 µl aliquot of the mock community was added to the PowerBead tubes containing 600 µl of CD1 buffer for DNA extraction. The quality of the extracted DNA was then evaluated by quantifying 16S gene copy numbers, assessing sequencing metrics, and comparing the observed taxonomic composition to the theoretical profile of the standard.

Additionally, the four sampling blanks collected at the time of sampling were processed and subjected to DNA extraction using the same protocol as described above for individual samples.

### 16S rRNA gene sequencing and sequencing data processing

Extracted DNA was subjected to quantitative polymerase chain reaction (qPCR) of the 16S rRNA gene to estimate 16S gene copy number in each sample, and to subsequently normalize the quantity of DNA for library preparation. Polymerase chain reaction (PCR) amplification of the V4 hypervariable region of the 16S rRNA gene was performed using a dual-indexing approach (Gohl et al. 2016). The PCR products were quantified with the PicoGreen dsDNA Assay Kit (Life Technologies, Carlsbad, CA), and normalized and multiplexed in equimolar amounts. Sample pools were spiked with 15% PhiX before being sequenced on the Illumina MiSeq platform (Illumina Inc., San Diego, CA) at the University of Minnesota Genomics Center. Illumina V3 cluster chemistry was used to obtain 2 × 300 bp paired-end reads. All samples were sequenced in the same pool on a single sequencing run. The sampling blanks, mock community, and extraction controls were processed and sequenced alongside the fecal samples.

#### Amplicon sequence variants table and taxonomic assignment

Raw paired-end reads were primer-trimmed using Cutadapt (Martin 2011), and all trimmed sequence reads were processed through the Division Amplicon Denoising Algorithm (DADA2 v.1.34) to generate amplicon sequence variants (ASVs) (Callahan et al. 2016). Briefly, forward and reverse reads were truncated at 240 bp and 170 bp length, respectively, using the *filterAndTrim* function. PhiX reads were discarded, and sequence reads were removed if they contained any ambiguous base and/or more than two expected errors. Filtered sequence reads were used to estimate an error rate using the *learnErrors function*, and then denoised using the *dada* function. Error corrected mated reads were merged into contigs using the *mergePairs* function with the default minimal overlap threshold. The ASV table was generated following the removal of chimeric contigs using the *removeBimeraDenovo* function. A summary table was generated to track the number of reads dropped at various steps of the analysis pipeline. ASVs were assigned taxonomy using the SILVA database (v.138.2) (Quast et al. 2013) and the *assignTaxonomy* function, and species were annotated using the *addSpecies* function. The final ASV count table and associated taxonomy file, along with the sample metadata, were used to create a phyloseq object using the *merged_phyloseq* function within the “phyloseq” package (v.1.50) (McMurdie and Holmes 2013).

The sequence data for the control mock samples were aligned to the ZymoBIOMICS reference database (https://s3.amazonaws.com/zymo-files/BioPool/ZymoBIOMICS.STD.refseq.v2.zip), which contains 16S rRNA reference sequences for all bacteria included in the mock community. Extraction efficiency was assessed by comparing the observed taxonomic composition of the mock communities to their theoretical distributions.

#### Removing likely contaminants and normalization of microbial counts

Likely contaminant ASVs were identified using the prevalence method as implemented in the “decontam” package (v.1.26) in R (Davis et al. 2018), and removed from downstream analysis. All negative controls, along with the library preparation negative controls consisting of molecular-grade water used for library preparation (*N* = 3), were also included in the prevalence method. After removing potential contaminant ASVs, a rarefaction curve was generated to visualize the number of observed ASVs as a function of sequencing depth. However, the data were not rarefied but, instead, normalized using CSS (normalization percentile = 0.5) with the “metagenomeSeq” package (v. 1.49.0) in R (Paulson et al. 2013) to account for uneven sequencing depth and to prevent potential data loss resulting from rarefying. Normalized ASV counts were then aggregated to the phylum, class, order, family, and genus level using the *tax_glom* function in phyloseq.

### Outcomes

The primary outcomes included alpha diversity metrics, beta diversity, and the relative abundance of microbial taxa between pooled workflows. Alpha diversity, representing the diversity within a sample, was assessed by calculating observed richness, Shannon diversity index, and Pielou’s evenness index. Beta diversity, which evaluates differences in overall microbial community composition between samples, was assessed using Bray–Curtis dissimilarity estimated from normalized ASV counts. Additionally, the number of raw sequencing reads and 16S copy numbers obtained from these workflows were analyzed. All analyses were performed separately for day-old and 20-day-old piglets, focusing on the aforementioned outcomes

### Statistical analysis

Before statistical analysis, four samples (three individual and one pooled DNA sample) were excluded due to low sequencing depth (<1000 raw reads). This filtering step reduced the total number of samples from 478 to 474 (255 individual samples, 80 fecal pools, 79 DNA pools, and 60 pen-floor samples). Data from sampling controls (*N* = 4), extraction controls (*N* = 7), and mock communities (*N* = 2) were summarized for quality control but not included in the statistical analyses due to their small sample sizes. To account for potential variation between litters and the nonindependence of piglets and pools within litters, litter ID (i.e. sow ID) was included as a random effect and litter size as a covariate in the regression models. Type III ANOVAs were used to test the significance of fixed effects in the mixed-effects models, accounting for both random effects and potential unequal sample sizes across different pool types. Since litters containing day-old piglets and 20-day-old piglets were housed in separate rooms, potential room effects on the outcomes were not assessed. All analyses were adjusted for multiple comparisons using the Benjamini–Hochberg (BH) method.

#### Evaluation of potential sequencing bias and 16S rRNA gene copy number between pool types

Differences in 16S qPCR copy numbers (log_10_ copies/µl) and number of raw reads generated from 16S rRNA gene sequencing were compared between pooled workflows using linear mixed models as implemented in the *lmer* function within the “lme4” and “lmerTest” v3.1–3 packages (Kuznetsova et al. 2017). The dataset was stratified by age group given large microbiome differences between 1-day and 20-day old piglets, and separate models were generated for each age group. In all models, litter size was included as a fixed effect, and litter ID (litter identity) was included as a random effect. The sex of the piglets, recorded at the time of sampling, was excluded from the models since it was not applicable after pooling. The significance of each fixed effect variable was determined using Type III ANOVA with a significance level set at *P* < .05, using the “car v3.1–2” package in R (https://cran.r-project.org/web/packages/car/index.html). Pairwise comparisons were conducted using the “emmeans” package (v.1.10.5) in R (https://github.com/rvlenth/emmeans).

#### Evaluation of difference of microbial community diversity (alpha) by pool types

Alpha diversity (i.e. richness, Shannon diversity, and Pielou’s evenness index) was calculated using ASV-to-genus-level (not normalized) counts with the alpha function in the “Microbiome” R package (v.1.28.0) (https://microbiome.github.io). Multivariable linear mixed-effects models were fitted to assess differences in each alpha diversity metric by pooled workflows (i.e. richness, Shannon diversity, and Pielou’s evenness index). Model covariates included litter size and sequencing depth (i.e. number of paired-end raw reads) as fixed effects, and litter ID as a random effect, as implemented in the *lmer* function in R. The significance of each fixed effect variable was determined using Type III ANOVA. Pairwise comparisons between pool types were conducted using the “emmeans” package. Additionally, rarefaction curves were used to compare microbial feature richness across pool types as a function of sequencing depth using the *ggrare* function within the “ranacapa” package (v. 0.1.0) in R (Kandlikar et al. 2018).

#### Overall microbial composition (beta-diversity) between pool types

To assess differences in beta diversity between samples, we calculated Bray–Curtis dissimilarities from a normalized ASV table using the *vegdist* function in the R package “vegan” (v.1.10.5) (Dixon 2003). Nonmetric multidimensional scaling (NMDS) plots and distance-based redundancy analysis (RDA) plots were generated using ggplot2 (Wickham 2016) to visualize microbial community patterns across all samples. We investigated variations in microbial composition by pooled workflows using permutational multivariate analysis of variance (PERMANOVA), implemented via the *adonis* function in the “vegan” R package (v. 2.6–4), with 999 permutations. The model included litter size, the number of paired-end raw reads, and litter ID. When a statistically significant result (*P* < .05) was observed from PERMANOVA, pairwise comparisons were performed using the *pairwise.adonis* function from the “pairwiseAdonis” R package (v. 0.4.1), applying the BH method to adjust for multiple comparisons.We also calculated beta-dispersion (i.e. distance to centroid) using the *betadisper* function from the “vegan” package and compared these values across pooled workflows and ages using ANOVA. All analyses were performed stratified by age and at each taxonomic level (i.e. ASV, phylum, class, order, family, and genus).

We also evaluated the potential impact of different normalization, transformation, and dimension reduction methods on beta diversity results. For this analysis, we stratified microbiome data by age (day-old and 20-day-old piglets) and compared the beta diversity of the different workflows using six different methods. These included: (i) raw data (i.e. nonnormalized and nontransformed ASV counts); (ii) rarefied data using the *even-depth* function in the “phyloseq” package in R; (iii) total sum scaling (TSS), in which raw ASV counts are divided by the total sum of counts for each sample; (iv) cumulative sum scaling (CSS), implemented using the “metagenomeSeq” package (v. 1.38.0) with the default normalization scaling factor; (v) one compositional data approach (CoDA), which employed Bayesian multiplicative replacement of zero counts (*method =“Bayes-Laplace”*) followed by centered log-ratio (CLR) transformation (i.e. Aitchison distance) using the “zCompositions” package (v. 1.4.0); and (vi) the Wrench normalization method, which accounts for group-specific normalization by incorporating pooled workflow variables using the “Wrench” package (v. 1.20) (Kumar et al. 2018) in R. Beta diversity analyses were performed using Bray–Curtis dissimilarity (calculated with the *vegdist* function in the “vegan” R package) and Aitchison distance (for CoDA-transformed data only) and visualized using two dimension reduction methods: principal coordinates analysis (PCoA) and NMDS implemented in the “vegan” R package. To assess differences in beta diversity by pooled workflow (*R*² values and associated *P*-values), we performed PERMANOVA for each dissimilarity metric derived from the normalization methods using the *adonis2* function implemented in the “vegan” R package. PERMANOVA analyses were adjusted for covariates litter ID, litter size, and raw reads, and were performed separately for day-old and 20-day-old piglets. Additionally, pairwise PERMANOVA analyses were used to compare pooled workflows using the *pairwise.adonis* function implemented in the “pairwiseAdonis” package R package.

#### Investigating the number of features (ASVs) shared across pooled workflows

To determine the number of features (i.e. ASVs) exclusive to individual samples versus shared across pooled workflows, we first calculated the unique ASVs associated with each workflow. We then used the *upset* function within the “UpSetR” package (v.1.10.5) in R to visualize the overlap of ASVs between workflows. Next, we assessed the prevalence (i.e. proportion of positive samples/proportion of samples in which a particular ASV is present) and abundance (i.e. summed across samples) of these unique ASVs. We considered ASVs to have low prevalence and abundance if they were present in ≤5% of samples and/or had a log_10_ abundance ≤2.

We estimated the proportion of potential sources contributing to microbial features in fecal and DNA pools using the SourceTracker model (Knights et al. 2011). This analysis aimed to identify microbial sources originating from individual samples and other potential sources, such as air swabs collected during sampling and extraction blanks prepared during DNA extraction. The SourceTracker model was applied separately to each litter, as pooling was conducted within individual litters (i.e. sow ID).

#### Investigating differences in relative abundance of microbial taxa between pool types and pig age

Differential abundance analysis was performed to determine phylum- and genus-level differences in relative abundance between day-old and 20-day-old piglet samples for each pooling workflow, and between pooled workflows for day-old and 20-day-old piglets separately. Differential abundance analysis between workflows considered only those phyla and genera present in at least one sample of each workflow. Normalized taxa counts were converted to integers prior to analysis. For each phylum or genus, a generalized linear-mixed model (GLMM) was fit to assess the difference in normalized taxa counts, with age or pooling workflow as a fixed effect, litter size as a covariate, raw reads as an offset, and litter ID as a random effect, using a negative binomial distribution (family = *nbinom2*) within the “glmmTMB” package (v1.1.10) in R. Estimated marginal means (EMMs) for pooling workflows were obtained, and pairwise comparisons between pooling workflows were performed using the “emmeans” package in R, with multiple comparison adjustment using the BH method. A separate differential abundance analysis was performed using the ANCOM-BC2 (analysis of compositions of microbiomes with bias correction) method with CLR transformed taxa counts (Lin and Peddada 2024). This additional analysis was performed to test differences (if any) in the abundance of phyla and genera between workflows, complementing the results obtained from the GLMM (negative binomial family) using normalized taxa counts. The analyses were stratified by day-old and 20-day-old samples, adjusting for covariates such as litter size and raw read counts, with litter ID included as a random effect to account for clustering within litters.

## Results

### Sequencing metrics and 16S rRNA gene copy number were similar across individual samples and fecal and DNA pools, but were significantly different from composite pen-floor samples

Illumina sequencing of the 16S rRNA gene amplicons yielded 17 million reads across all libraries [thousands (k) range: 0.04–64 k per sample] with a mean quality score of 31 (range: 28–33 per sample). This included individual samples (*N* = 258), fecal and DNA pools (*N* = 80 each), composite floor samples (*N* = 60), sampling controls (*N* = 4), extraction blanks (*N* = 7), and mock communities (*N* = 2) (Fig. S2A–D). After excluding three individual libraries and one pooled DNA library that yielded ≤1 k reads, the average number of raw sequencing reads was similar between samples obtained from day-old [emmeans/EMM, 95% CI: 34.4 (33.2–36.5)] and 20-day-old piglets [36.8 (35.1–38.5)] (Type-III ANOVA *P* = .09). Further, no significant association was observed between average raw reads and litter size [35.8 (34.7–37.0), Type-III ANOVA *P* = .743]. Additionally, no difference was observed in the average raw reads generated from samples obtained from the two different Qiacubes used for extractions [35.3 (34.1–36.5), 35.8 (34.4–37.2), *P* = .571], or extraction date (*P* = .062).

The average number of raw sequences was similar across individual samples, fecal pools, and DNA pools for both day-old and 20-day-old piglets, although DNA pools yielded a slightly higher average number of raw reads. For day-old piglets, the average number of raw sequences for individual samples, fecal pools, and DNA pools was 34.3 k (95% CI: 31.8–36.8 k), 35.1 k (95% CI: 31.6–38.6 k), and 37.2 k (95% CI: 33.7–40.7 k), respectively. In contrast, composite floor samples from these piglets yielded 28.7k reads (95% CI: 24.8–32.6 k), which was significantly lower than the individual, fecal, and DNA pools (pairwise *P* < .05). For 20-day-old piglets, the average number of raw sequences generated for individual samples, fecal pools, and DNA pools was 35.9 k (95% CI: 33.8–38.0 k), 36.k (95% CI: 33.5–39.7 k), and 39.4 k (95% CI: 36.3–42.5 k), respectively. Composite floor samples collected from the 20-day-old pens yielded 37.7 k reads (95% CI: 34.2–41.3 k), with no statistically significant difference compared to individual, fecal, and DNA pools (pairwise *P* = .783, *P* = .959, and *P* = .887, respectively).

The total number of raw reads derived from sampling controls and extraction blanks was 17 k (ranging from 0.2 to 7.9 k) and 8.3 k (ranging from 0.3 to 3.4 k), respectively. As expected, the negative controls formed separate clusters from the actual samples, thereby indicating distinct microbial community compositions (Fig. S2G).

After quality filtering, merging paired-end reads, and chimera removal, an average of 23.4 k ± 7.6 k (73%), 22.2 k ± 7.3 k (69%), and 21.3 k ± 7.0 k (67%) of sequenced reads remained, respectively (Fig. S3, Table S3). Across all samples, 3859 ASVs were identified. Of these, 77 ASVs (∼0.1% of total filtered reads) were identified as likely contaminants by the prevalence method, and removed from subsequent analyses. After the removal of these potential contaminants, ASVs were classified into 29 phyla (encompassing 99% of ASVs), 49 classes (98%), 101 orders (96%), 173 families (87%), 467 genera (67%), and 414 species (19%) (Fig. S4).

The average 16S rRNA gene copy number, as determined by the number of 16S rRNA gene copies per µl via quantitative PCR, was not statistically significantly different between individual samples, fecal and DNA pools from day-old and 20-day-old piglets (Figs S2E, F, and S5). Day-old piglet individual samples had an average log_10_ concentration of 6.5 (95% CI: 6.2–6.8), while fecal and DNA pools had averages of 6.6 (95% CI: 6.3–7.0) and 6.4 (95% CI: 6.0–6.8), respectively. Composite floor samples from these piglets had a significantly lower average 16S rRNA gene copy number than individual, fecal, and DNA pools (pairwise comparison *P* < .01). Twenty day-old piglets’ individual samples had an average log_10_ copy number of 6.6 (95% CI: 6.4–6.7), while fecal pools had a similar average [6.5 (95% CI: 6.3–6.8)] and DNA pools were slightly higher [6.8 (95% CI: 6.6–7.0)]. However, composite floor samples for this age group exhibited a significantly higher average copy number compared to the other pool types (pairwise comparison *P* < .01) (Fig. S5). As expected, sampling controls (*N* = 4) and extraction blanks (*N* = 7) showed lower log_10_ values for 16S rRNA gene copies, with means (SD) of 0.8 ± 0.4 and 0.6 ± 0.4, respectively.

Two Zymo mock communities were used as positive controls and yielded 24.3 k and 33.3 k raw reads, with mean quality scores ≥33 and log_10_ 16S rRNA gene copy numbers of 5.08 and 5.44, respectively. We detected taxa with a theoretical composition as low as 0.07% (e.g. *Salmonella* spp.), indicating our ability to detect low-abundance taxa at the sequencing depth used in this study (Fig. S2H and I).

### The fecal microbiome recovered from DNA and fecal pools was similar to individual samples, but different from composite pen-floor samples

**Figure 2.**
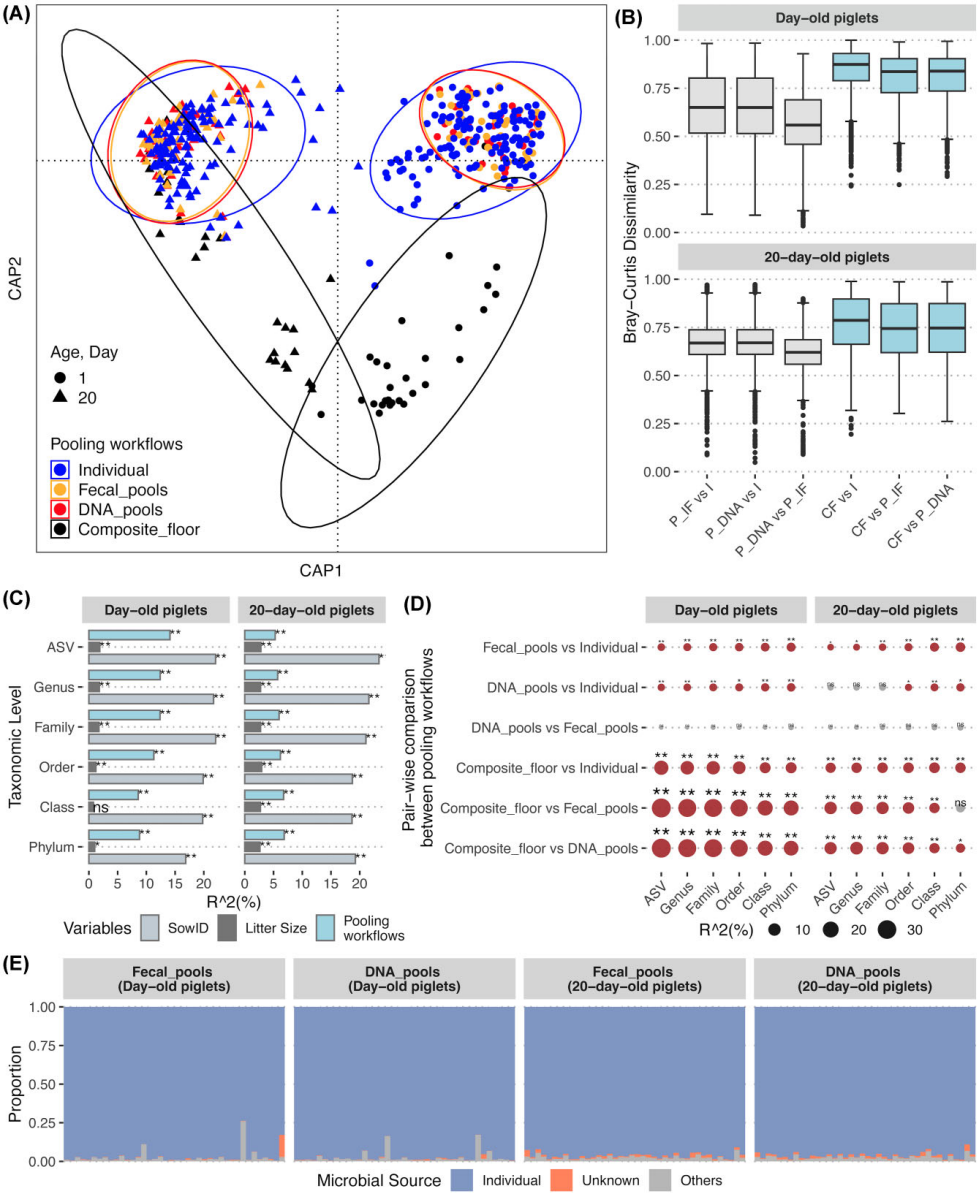
Microbial community composition across pool types, stratified by pig age. (A) Distance-based RDA of Bray–Curtis dissimilarities between microbial features (ASVs) in samples from all pool types from day-old and 20-day old piglets. (B) Box plots representing pairwise Bray–Curtis dissimilarities between individual samples and fecal or DNA pools (gray), and between composite floor samples and individual samples, fecal, or DNA pools (blue). I—individual, P_IF and P_DNA—fecal and DNA pools, respectively, CF—composite pen-floor. (C) *R*²-values from PERMANOVA. (D) Pairwise comparisons of pool types, with size of dot proportional to *R*² value. For (C) and (D), asterisks indicate BH-adjusted *P*-values as follows: **P < .01, *P < .05, ns (not significant) *P* > .05. (E) SourceTracker results showing the likely source of ASVs in fecal and DNA pools.

RDA based on Bray–Curtis dissimilarities showed that samples from day-old and 20-day-old piglets clustered distinctly, as expected (PERMANOVA *R*² = 26%, *P* = .001, Fig. 2A). Within each age-specific cluster, DNA and fecal pools exhibited tight clustering, and individual samples overlapped with these pooled libraries, suggesting similarities in their overall microbial composition. In contrast, composite floor samples showed a clear separation from individual samples, fecal and DNA pools, indicating differences in microbial composition (Fig. 2A, Fig. S6). The beta-dispersion analysis at the ASV level showed that the average distance to the centroid was higher for individual samples compared to pooled and composite pen-floor samples in both day-old (Individual: 0.50, DNA pools: 0.40, fecal pools: 0.40, composite pen floor: 0.40, ANOVA *P* < .01) and 20-day-old piglets (Individual: 0.49, DNA pools: 0.44, fecal pools: 0.43, composite pen floor: 0.52, ANOVA *P* < .01) (Fig. S6). This suggests that microbial composition was more homogenous/reduced variability within pooled samples compared to individual samples.

Omnibus and subsequent pairwise comparison testing with PERMANOVA of Bray–Curtis dissimilarity further confirmed significant differences in the overall fecal microbial community composition by pooled workflow, except between DNA and fecal pools. These differences were observed across all taxonomic levels analyzed and for both age groups (Fig. 2C and D, Fig. S6). Variation partitioned to pooled workflow ranged from 8% to 14% in day-old piglets and 5% to 7% in 20-day-old piglets, from phylum to ASV level (Fig. 2C). In both age groups, litter ID accounted for ~16%–23% of the variation, while litter size contributed <3%, across all levels of the taxonomy (Fig. 2C). Although we observed statistically significant differences in microbial composition between individual samples and both DNA and fecal pools, the amount of variation partitioned was low (*R*² ≤ 5% across all taxonomic levels) (Fig. 2D). Using the SourceTracker modeling approach, we found that the majority of ASVs detected in pooled (either fecal or DNA) samples were likely sourced from individual samples. In both day-old and 20-day-old piglets, microbial features likely originating from individual sources constituted an average of 95% (range: 73%–99%) of all features in both DNA and fecal pools (Fig. 2E), while the proportion of features in the DNA and fecal pools originating from “unknown sources” was very low (<1%). Contributions from sampling blanks (categorized as “others”) were very low, i.e. typically <5% of all features.

The microbial composition in composite floor samples was significantly different from that in individual samples, fecal pools, and DNA pools (all possible pairwise PERMANOVA tests, *P* < 0.05). These differences were consistent across both age groups and all taxonomic levels (Fig. 2D). Additionally, the average Bray–Curtis distance was lower between individual samples and both fecal and DNA pools than between composite floor samples and individual, fecal, and DNA pools (Fig. 2B, Fig. S7). This further suggests that the microbial communities recovered in DNA and fecal pools are more similar to each other and to individual samples than composite pen-floor samples.

These patterns in beta diversity remained remarkably consistent when different normalization/transformation and dimension reduction approaches were used (Figs S8 and S9). In all cases, beta diversity differed between the pen-floor samples and the other three workflows, for both day-old (*R*² = 11%–17%) and 20-day-old (*R*² = 3%–5%) piglets. Pairwise PERMANOVA analyses further supported these findings, revealing similar differences between pooled workflow results across all analytical methods (Fig. S10). While the overall *R*² values and pairwise differences between pooling workflows remained similar, some variations in clustering patterns were observed between PCoA and NMDS ordination when using methods such as CLR and Wrench. Bray–Curtis dissimilarities calculated from raw ASV counts, rarefied data, and TSS and CSS normalization methods exhibited similar ordination patterns in both PCoA and NMDS. However, the Bayesian multiplicative replacement with CLR transformation showed higher variability in beta diversity amongst the pen-floor samples.

### Pooled and individual samples had similar alpha diversity, which differed from that of composite floor samples

**Figure 3.**
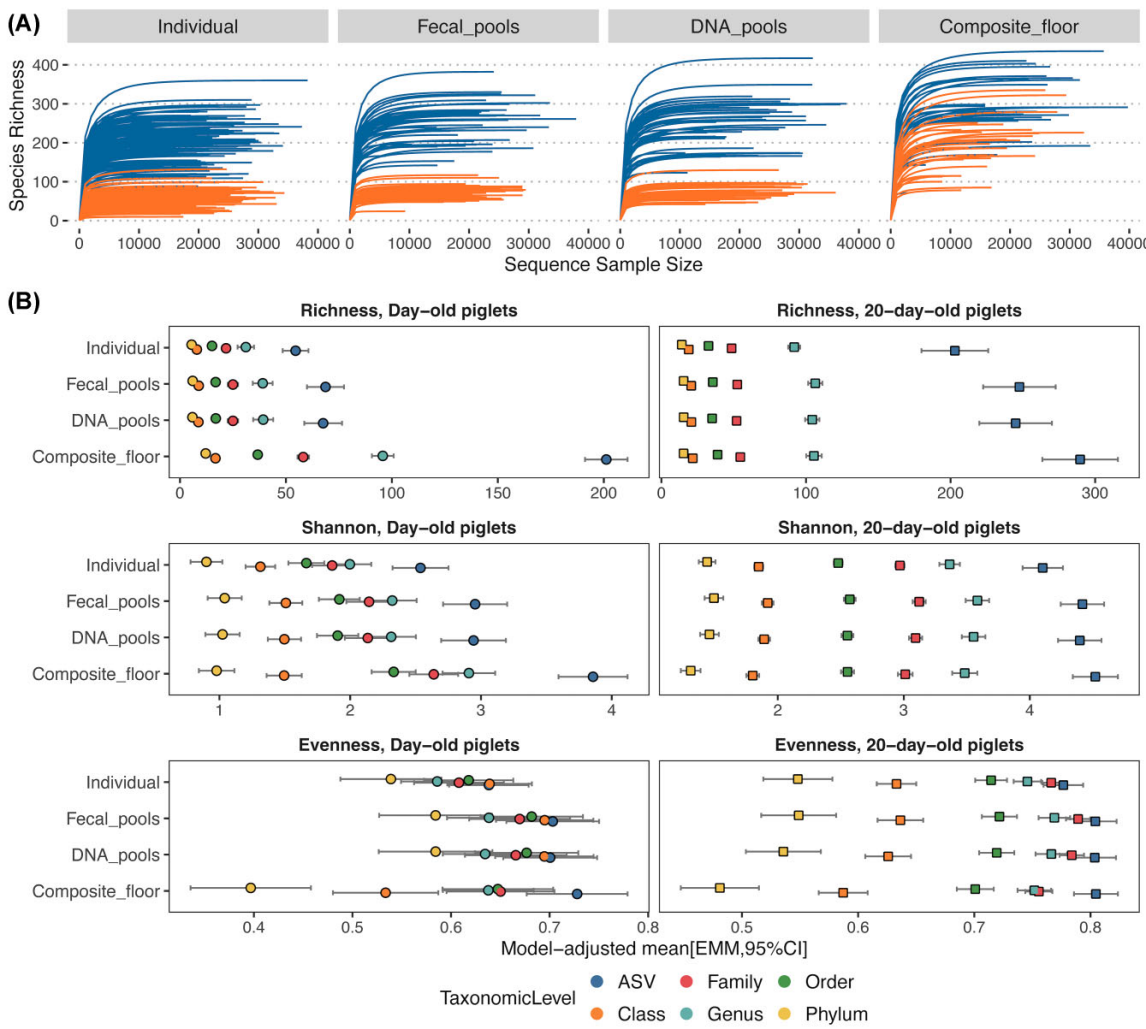
Estimates of fecal microbial community alpha diversity across pool types. (A) Rarefaction curves showing the number of unique microbial features detected by sequencing depth, stratified by day-old piglets (orange lines) and 20-day-old piglets (blue lines). (B) Alpha diversity estimates (i.e. richness, Shannon diversity, and Pielou’s evenness), stratified by pig age and taxonomic level. Points and whiskers represent model-adjusted means and 95% confidence intervals, respectively, obtained from multivariable mixed models.

Rarefaction analysis showed that a sequencing depth of ~10 000 reads per sample effectively captures the microbial richness in individual samples, as indicated by the leveling off of richness at this depth (Fig. 3A). Both fecal and DNA pools displayed similar patterns, achieving comparable richness levels at this sequencing depth. In contrast, composite floor samples reached their highest richness plateau at a similar sequencing depth.

Alpha diversity analysis revealed that average ASV richness was lower in individual samples compared to fecal or DNA pools. For example, in day-old piglets, individual samples had an average richness of 54.5 ASVs (95% CI: 48.7, 60.6), compared to fecal pools [68.7 (95% CI: 59.9, 77.4)] and DNA pools [67.6 (95% CI: 58.6, 76.5)]. In 20-day-old piglets, average ASV richness was higher than in day-old piglets, as expected, and the differences in ASV richness between workflows were more noticeable compared to day-old piglets. Specifically, average ASV richness for individual samples from 20-day-old piglets was 202.9 (95% CI: 179.8, 226.1), compared to 247.6 for fecal pools (95% CI: 222.5, 272.6) and 245 for DNA pools (95% CI: 219.9, 270.1). Similar results were observed for Shannon diversity and Pielou’s evenness, with individual samples showing modestly lower diversity and evenness compared to fecal and DNA pools (Fig. 3B). Similar results were observed across various taxonomic levels (Fig. 3B). In contrast, composite floor samples from both age groups demonstrated significantly higher average richness, Shannon diversity, and evenness compared to individual samples and DNA and fecal pools (all pairwise *P* < .05).

A particularly noteworthy finding was that alpha diversity metrics did not differ between fecal and DNA pools across all examined taxonomic levels and both age groups. For instance, in day-old piglets, the estimated mean difference between fecal and DNA pools in ASV richness was 1.1 (95% CI: −14.0, 16.2), in Shannon diversity 0.01 (95% CI: −0.3, 0.3), and in evenness 0.002 (95% CI: −0.06, 0.07). Similar results were observed in 20-day-old piglets, with the estimated mean differences being 2.6 in ASV richness (95% CI: −24.6, 29.8), 0.02 in Shannon diversity (95% CI: −0.15, 0.2), and 0.0004 in evenness (95% CI: −0.02, 0.01). Further, we observed consistency in these findings across all analyzed taxonomic levels.

In summary, these results demonstrate that alpha diversity metrics between fecal and DNA pools are similar across various taxonomic levels and in both day-old and 20-day-old piglets. This suggests that the methodologies employed for pooling—whether from fecal pools or extracted DNA—do not significantly affect the inferred representation of microbial alpha diversity. Further, alpha diversity metrics obtained from pools (either fecal or DNA) were only marginally higher than those obtained from individual samples, while the largest differences were observed when comparing either individual samples or pools to composite floor samples.

### High prevalence, high abundance features were recovered by all pooling workflows, while low prevalence and low abundance features were more likely to be captured in individual versus pooled samples

**Figure 4.**
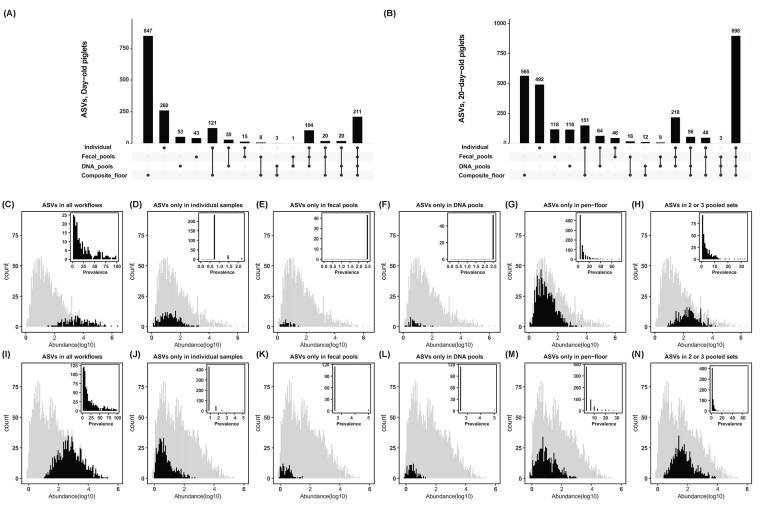
ASV distributions across pooled workflows. The UpSet plot shows shared and unique ASVs across pooled workflows for (A) day-old and (B) 20-day-old piglets. Distribution of ASVs (abundance and prevalence) across pooled workflows: (C–H) day-old and (I–N) 20-day-old piglets. The *x*-axis represents the abundance of ASVs (summed across samples), with gray bars representing ASV abundance across all workflows and black bars representing ASV abundance only in the given pooled/workflow sample set. The *y*-axis shows the count of ASVs. Inset plots depict the distribution of ASV prevalence (%) within the given pooled/workflow sample set. “ASVs in all workflows”—ASVs present in all pooled workflows, including individual samples, fecal, DNA pools, and composite floor sample sets. “ASVs only in individual samples”—ASVs found only in individual samples and absent in all pooled sample types (composite_floor, DNA pools, fecal pools). “ASVs only in fecal pools”—ASVs found only in fecal pool sample set and absent in individual, composite floor, and DNA pool sample sets. “ASVs only in DNA pools“—ASVs found only in DNA pool sample set and absent in individual, composite floor, and fecal pool sample sets. ”ASVs only in pen-floor“—ASVs found only in composite floor sample set and absent in individual, DNA pool, and fecal pool sample sets. “ASVs in 2 or 3 pooled sets”—ASVs detected in two or three of the pooled sample sets.

Of the 1736 total ASVs found across all samples from day-old piglets, 211 (12.1%) were detected in at least one sample within each of the four workflows, whereas 104 (6.0%) were detected in at least one sample from each of individual samples, fecal, and DNA pools (Fig. 4A). In 20-day-old piglets, 31.9% (898/2814) of the total detected ASVs were detected in at least one sample across all workflows, and 7.7% (218/2814) were detected in at least one sample from each of individual samples, fecal, and DNA pools (Fig. 4B). In day-old pigs, nearly 50% of all ASVs (847/1736) were only detected in pen-floor samples, while this proportion was much lower in 20-day old pigs at 20.1% (565/2814). These results suggest that composite floor samples may capture more of the ASVs present in 20-day old individual piglets as compared to day-old piglets. When we further analyzed individual, fecal, and DNA pools (excluding the composite floor samples), we found that 35.4% (315/889) and 49.6% (1116/2249) of ASVs were common between individual samples, DNA and fecal pools in day-old and 20-day-old piglets, respectively (Fig. S11).

In both age groups, ASVs found within only one of the four workflows were typically of low abundance and low prevalence. For example, in day-old and 20-day-old piglets, a total of 260 and 492 ASVs were detected only within the individual sample set (Fig. 4A and B), and all of these ASVs were present in <5% of individual samples, and with very low relative abundance (Fig. 4D and J). In day-old pigs, the average log_10_ abundance of the 260 ASVs found only in individual samples was 1.3 (range: 0.13–3.23), and with an average prevalence within the individual sample set of 0.8% (range: 0.73%–2.2%) (Fig. 4D). For samples from 20-day old pigs, the 492 ASVs found only in the individual sample set had an average log_10_ abundance of 0.7 (range: 0.02–2.7) and an average prevalence within the individual sample set of 0.9% (range: 0.83%–4.9%) (Fig. 4J).

We also identified ASVs in fecal and DNA pools that were not recovered in either the individual or the composite floor sample sets, encompassing 43 and 53 ASVs in fecal and DNA pools, respectively, for day-old piglets; and 118 and 116 ASVs, respectively, for 20-day-old piglets. All of these ASVs were characterized by low prevalence (≤5% of fecal or DNA pool samples) and low abundance (log_10_ ≤2) (Fig. 4E, F, K, and L).

In contrast, composite floor samples had a higher count of unique ASVs that were not found in individual, fecal, or DNA pools, across both age groups. Like the “individual-only ASVs”, these “composite-only ASVs” exhibited low mean abundance in both the day-old and 20-day old pigs [mean 1.2 (range: 0.09–3.6) for day-old pigs, and 1.0 (range: −0.10–2.9) in 20-day old pigs]. However, unlike the ASVs found only in the individual and pooled sample sets, the ASVs found only in the composite floor samples had higher prevalence within the composite samples, with a mean of 8.6% for day-old pigs (range: 3.3%–73.3%) and 5.7% in 20-day old pigs (range: 3.3%–33.3%).

Conversely, ASVs that were detected across all four of the workflows typically had higher prevalence and relatively higher abundance. Specifically, the abundance and prevalence of these common ASVs were 3.6 (range: 1.5–6.4) and 25% (range: 1.6%–98.8%) in day-old piglets, respectively; and 2.8 (range: 1.06–5.3) and 22.5% (range: 1.7%–98.7%) in 20-day-old piglets, respectively (Fig. 4C and I). Similar results of higher prevalence but not abundance was observed for ASVs detected in two or three of the pooled sample sets (Fig. 4H and N), with mean abundance of 2.3 (range: 0.9–4.20) and mean prevalence of 4.2% (range: 0.8%–32.8%) in day-old piglets, and mean abundance of 1.7 (range: 0.4–3.9) and mean prevalence of 3.1% (range: 0.8%–65.4%) in 20-day-old piglets (Fig. 4H and N).

These findings suggest that ASVs with higher abundance and prevalence are recovered from all workflows, i.e. individual, fecal, DNA pools, and composite floor samples, whereas ASVs with low prevalence and low abundance tend to be recovered in only one of the four workflows.

**Figure 5.**
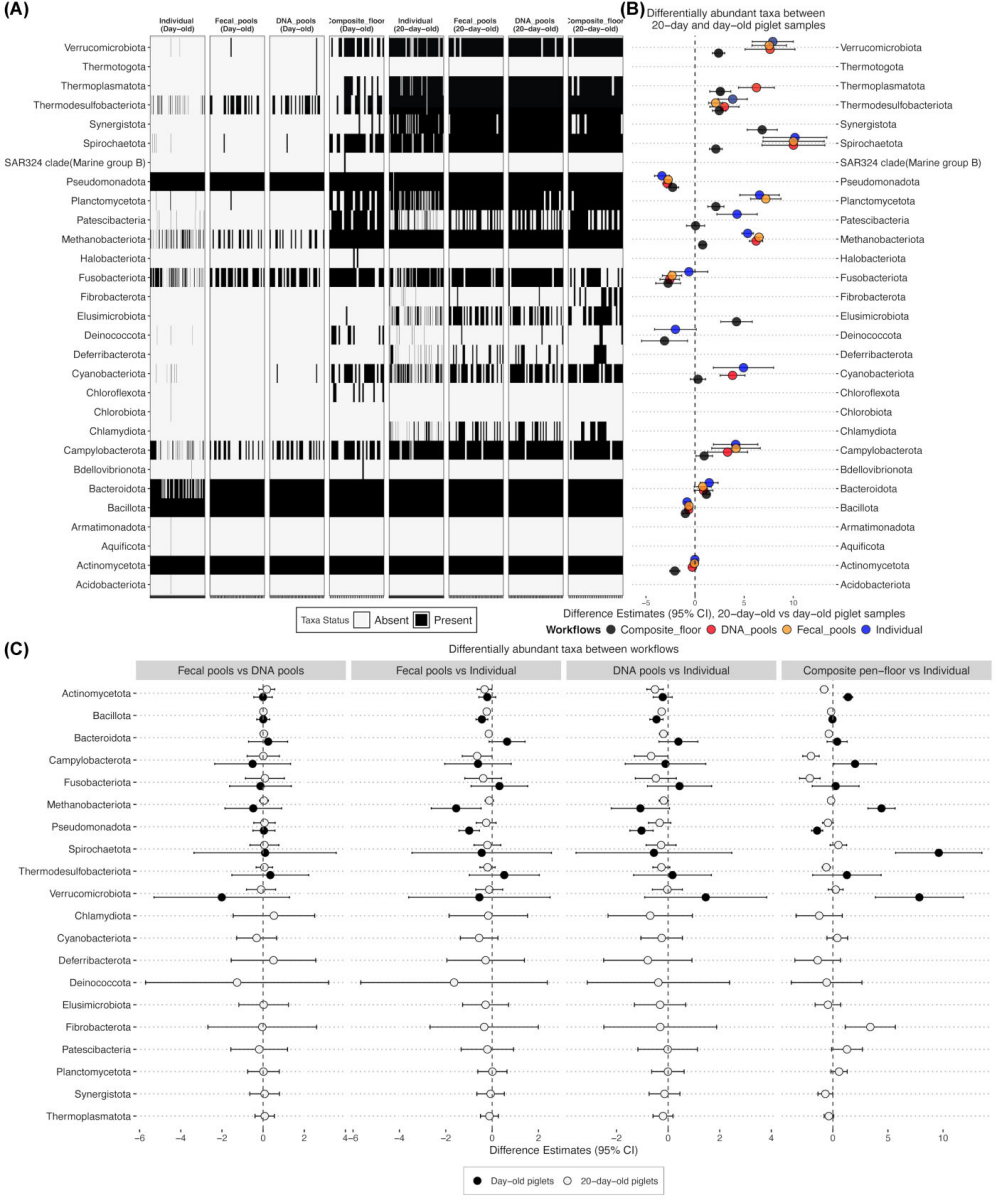
Differential detection and abundance analysis. (A) Heatmap depicting the presence (black) or absence (white) of phyla (*y*-axis) across individual samples (*x*-axis), stratified by workflow (individual, fecal pool, DNA pool, and composite floor) and age (day-old and 20-day-old). (B) Differential abundance analysis of phyla between 20-day-old and day-old piglets, stratified by workflow. Points represent estimates, and error bars indicate the 95% confidence intervals of the estimates. Positive estimates indicate higher abundance in 20-day-old piglets compared to day-old piglets. Phyla with 95% confidence intervals that do not overlap zero are considered significantly differentially abundant. Phyla present only in either day-old or 20-day-old samples within specific workflows were excluded from the visualization. (C) Differential abundance analysis of phyla (*y*-axis) changes between workflows, stratified by age group (filled black circles = day-old piglets, gray circles = 20-day-old piglets). Error bars (95% CI) that do not overlap zero indicate significantly differential phyla. Note that the taxa with gray circles in panel C were only identified in 20-day-old piglets.

By annotating ASVs at the phylum level, we found that several phyla were present in individual samples but absent in both fecal and DNA pool sets (Fig. 5A). These included *Acidobacteriota, Acquificota, Armatimonadota, Chloroflexota, Chlorobiota, Deinococcota*, and *Patescibacteria* in day-old piglet samples. The absence of these phyla in pooled samples could be due to limitations in detecting low-abundance taxa, as the relative abundance of these phyla was ≤1%. On the other hand, phyla such as *Thermotogota* and *Thermoplasmatota* were detected only in DNA pools and were absent in individual and fecal pool sets, while *Synergistota, SAR324 clade, Halobacteriota*, and *Elusimicrobiota* were found only in composite pen-floor samples from day-old piglets. In 20-day-old piglets, all detected phyla were present in at least one sample from individual, fecal pools, DNA pools, and composite pen-floor sample sets. In both day-old and 20-day-old piglet samples, *Bacteroidota, Bacillota, Actinomycetota, Fusobacteriota*, and *Pseudomonadota* were prevalent across all samples, suggesting that these phyla can be recovered using any of the four workflows.

### Pooling method can selectively affect the detection, and relative abundance estimate of specific microbial taxa

Of the 29 phyla identified, 17 were differentially abundant (DA) between day-old and 20-day-old piglet samples across all four workflows (BH Adj. *P* < .05) (Fig. 5). Among these, 11, 9, 10, and 14 phyla were DA when analyzing only the individual, fecal pools, DNA pools, and composite pen-floor samples, respectively. The majority of these DA phyla (12/17) were more abundant in 20-day-old piglets compared to day-old piglets (Fig. 5B). For example, across all four workflows, *Methanobacteriota, Thermodesulfobacteriota, Verrucomicrobiota*, and *Spirochaetota* were significantly more abundant in 20- versus day-old piglets (difference in GLMM estimates ranged from 0.8 to 10.1). Additionally, when using individual, fecal, and DNA pools, *Campylobacterota* was more abundant in 20-versus day-old piglets (GLMM estimate differences ranging from 3.3 to 4.1), while using composite-floor samples indicated that *Synergistota* was more abundant in 20- versus day-old piglets. In contrast, some phyla were significantly more abundant in day-old piglets as compared to 20-day old piglets. Across all four workflows, *Pseudomonadota* and *Bacillota* were more abundant in day-old versus 20-day-old piglets (GLMM estimate differences ranging from 0.6 to 2.3). Within the DNA pools, fecal pools, and composite pen-floor sample sets, *Fusobacteriota* was also significantly more abundant in day-old piglets (GLMM estimates ranging from 2.7 to 3.4). At the genus level, as expected, numerous genera were DA when comparing 20-day-old and day-old piglet samples. A total of 90 genera were DA in individual samples (68 with higher and 22 with lower abundance), 63 in DNA pools (45 higher and 18 lower), 61 in fecal pools (43 higher and 18 lower), and 94 in composite samples (56 with higher abundance and 38 with lower abundance).

Differential abundance analysis was also used to identify phyla and genera that were recovered at significantly higher or lower abundances in specific workflows within day-old and 20-day-old piglet samples. For this analysis, only phyla and genera detected in at least one sample from each of the four workflows were included. Based on this analysis, three phyla (out of 10) in day-old piglets and two phyla (out of 20) in 20-day-old piglets were significantly DA between pooled samples (fecal or DNA pools) and individual samples (Fig. 5C). In day-old piglets, *Bacillota* had significantly lower abundance in pooled samples compared to individual samples [DNA pools: −0.45 (95% CI: −0.70 to −0.19); fecal pools: −0.45 (95% CI: −0.69 to −0.18)]. *Pseudomonadota* also significantly lower abundance in both DNA pools [GLMM estimate: −1.02 (95% CI: −1.47 to −0.57)] and fecal pools [estimate: −0.98 (95% CI: −1.43 to −0.54)] compared to individual samples, while *Methanobacteriota* were less abundant in fecal pools compared to individual samples [−1.55 (95% CI: −2.62 to −0.47)]. In 20-day-old piglets, the pattern continued with *Bacillota* showing lower abundance in DNA pools [estimate: −0.25 (95% CI: −0.39 to −0.11)] and fecal pools [estimate: −0.23 (95% CI: −0.37 to −0.10)] compared to individual samples. Additionally, *Actinomycetota* were less abundant in DNA pools compared to individual samples [estimate: −0.50 (95% CI: −0.82 to −0.19)] (Fig. 5C). At the genus level, most genera were less abundant in DNA and fecal pools compared to individual samples, although the differences in estimates were modest (ranging from −2.80 to −0.70). For example, eight and six genera (out of 110 total) in day-old piglet samples and three genera (out of 199 total) in 20-day-old piglet samples exhibited significant differences in abundance between individual samples and DNA pools, and between individual samples and fecal pools, respectively. All these DA genera were less abundant in pools compared to individual samples.

Differential abundance analysis comparing DNA and fecal pool samples showed that nearly all phyla and genera had similar relative abundances. No phylum or genus was significantly DA between fecal pools and DNA pools in either day-old or 20-day-old piglet samples (Figs S12 and S13).

The comparison of composite pen-floor and individual samples revealed differences in the abundance of several phyla and genera. In day-old piglets, *Pseudomonadota* was less abundant in composite floor samples [−1.42 (95% CI: −1.93 to −0.91)], while four phyla, including *Actinomycetota, Methanobacteriota, Spirochaetota*, and *Verrucomicrobiota*, were significantly more abundant in composite floor samples compared to individual samples (GLMM estimate differences ranging from 1.3 to 9.3). In 20-day-old piglets, five phyla (*Actinomycetota, Bacteroidota, Campylobacterota, Fusobacteriota*, and *Thermodesulfobacteriota*) were less abundant in composite floor samples (estimate differences ranging from −2.1 to −0.36), while *Fibrobacterota* was the only phylum with higher abundance in composite floor samples [0.88 (95% CI: 1.12–5.67)] (Fig. 5C). At the genus level, we identified 48 and 49 DA genera in day-old and 20-day-old piglets, respectively. Of these, 35 genera in day-old piglets and 18 genera in 20-day-old piglets showed higher abundance in composite floor samples compared to individual samples (Figs S12 and S13).

An additional differential abundance analysis using ANCOM-BC2 with CLR-transformed counts also identified several DA taxa between workflows. The largest number of DA phyla and genera were observed when comparing composite pen-floor and individual samples. This observation was consistent with the results obtained using the GLMM approach. However, the ANCOM-BC2 method identified fewer statistically significant DA phyla and genera between workflows compared to the GLMM. In day-old piglets, only *Methanobacteriota* and *Actinomycetota* were significantly more abundant in composite pen-floor samples compared to individual samples, with no other phyla displaying significant differences between any other workflows. These two phyla were also identified as DA between composite pen-floor and individual samples using the negative binomial GLMM. At the genus level, 27 and 9 genera were DA between individual and composite pen-floor samples in day-old and 20-day-old piglet samples, respectively (Table S4). It is important to highlight that both differential abundance analysis methods yielded the same results when comparing fecal pools and DNA pools, with no statistically significant difference at either the phylum or genus levels in both day-old and 20-day-old samples.

## Discussion

Pooled testing offers an efficient method for large-scale studies by combining individual samples into a group or pool for analysis (Cleary et al. 2021). In this study, we investigated the impact of various pooling methods on the observed fecal microbiome profile of preweaned piglets. Our results showed that pooling raw feces or extracted DNA from individual samples within a litter and sequencing the resulting pools can provide a reasonable approximation of the litter-level microbiome profile of individual piglets within the litter. While these pooling methods may underestimate the detection of some low-abundance and/or low-prevalence taxa, the overall microbial diversity and composition were comparable to those obtained from individually collected samples. The effectiveness of these pools can be attributed to the shared pen environment among piglets in the same litter, which may foster a homogenous microbiome among littermates (Fredriksen et al. 2022). SourceTracker analysis further supports this conclusion, showing that pooled samples retain the majority of microbial sources present in individual samples. In contrast, pen-floor composite samples generate very different diversity profiles than individual samples, and should not be used as a proxy for microbiome dynamics of the preweaned piglets within the pen. These results contrast with a previous study, which suggested that composite floor fecal samples represent well the fecal microbiome in postweaned pigs (Munk et al. 2017). The discrepant findings likely arise from our study’s focus on preweaning piglets, whereas Munk et al. (2017) used weaned pigs. Specifically, we hypothesize that the presence of the sow within the pen environment of the preweaned pigs significantly influenced the microbial profile obtained from the pen-floor composite samples. This hypothesis is supported by the fact that the diversity metrics of composite pen-floor samples were more divergent from the day-old pig individual samples than the 20-day-old pig individual samples (Figs 2 and 3). Day-old pigs generally produce very little fecal material compared to the sow, which could explain the larger difference in microbial profiles for this age group. In contrast, 20-day-old pigs may contribute more fecal material to the pen environment, and thus their fecal material may be better represented within the microbiome of the composite pen-floor sample—even with the confounding influence of the sow’s feces. These results, along with those of Munk et al. (2017), demonstrate the importance of host population structure and management practices in determining the utility of composite samples for microbiome analysis.

Importantly, pig pen-floors likely harbor diverse microbes, including those from sow feces and various types of animal waste, as well as contaminants from the footwear of swine workers. Consequently, composite floor samples may accumulate diverse microbial sources over time, differing from what is typically found within young piglets (Chen et al. 2022). While sow feces were not collected in this study, future research incorporating sow fecal samples alongside individual piglet and pen-floor samples could provide additional insights into the microbial composition of the pen-floor microbiome. Combined with and compared to previous findings, our results suggest that careful consideration is necessary when using pen-floor samples for fecal microbiome analysis in preweaned piglets.

Depending on the purpose and available resources, either fecal or DNA pooling can be a cost-effective approach in litter-level fecal microbiome studies of preweaned piglets. Microbial profiles, including diversity, composition, and relative abundance, were largely similar between fecal and DNA pools, suggesting that either method could be considered, depending on circumstances. Fecal pools may be preferable to reduce the number of DNA extractions, particularly if cost is a constraining factor. However, fecal pooling requires careful aliquoting and homogenization of the material, which can be labor-intensive and time-consuming, particularly when trying to minimize cross-contamination during pooling. DNA pools, on the other hand, are generated after individual DNA extractions have been performed. This reduces the need for raw fecal processing for fecal pools (i.e. aliquoting of individual samples during the initial preparation steps), but it increases extraction costs due to the requirement for individual DNA extractions prior to pooling. Conversely, individually extracted DNA can be stored and used for other purposes, such as PCR for individual targets. However, both fecal and DNA pooling reduce the number of individual samples that need to be processed and/or sequenced, thereby reducing cost and labor. For instance, in our study with a fixed number of pools per litter/group, fecal pools can reduce the number of samples processed and sequenced by ~4-fold. In our experience, one person can process and extract DNA from 48 individual samples in 1 day using one Qiacube (i.e. 12 samples per batch × 4 batches = 48). This means that for 20 litters, each with on average 13 piglets (260 samples), it would take a total of 22 runs (or 4 batches/day = 6 days). However, with fecal pools (assuming 4 pools per litter, 20 × 4 per litter = 80), the same person can complete the DNA extraction in 7 runs (1.5 days). Additionally, both fecal and DNA pooling can reduce costs in large-scale microbiome studies by lowering expenditures on reagents and/or sequencing costs due to fewer library preparation reactions. If researchers choose to reallocate those cost savings toward achieving greater sequencing depth, pooled samples would yield more sequencing reads. This increased sequencing depth could enhance the likelihood of capturing increased taxonomic diversity through the detection of low abundance taxa (Gweon et al. 2019).

Although increasing the number of individual samples represented in fecal or DNA pools can further reduce costs, it is unknown how this modification would impact the representativeness of the resulting pools. Here, we considered a simple pooling strategy that is easy to implement for high-throughput sample processing. Specifically, four fecal or DNA pools were created per litter, regardless of litter size, with equal volumes of raw feces used to create fecal pools and equal volumes of DNA (irrespective of DNA concentration) used for DNA pools. For the DNA pools, this approach allowed us to sidestep the need to measure DNA concentration for each sample and subsequently adjust the input volume for each sample, which is costly and time-consuming. For fecal pools, an alternative approach would be to use equal-weight pooling; however, the use of equal-volume allowed us to sidestep the need to weigh out each individual fecal sample, which would increase both the workflow duration and the potential for sample contamination. Future studies could investigate the effect of equal-volume versus equal-concentration pooling for DNA pools, and equal-volume versus equal-weight pooling for fecal pools, by performing a head-to-head comparison on biological and technical replicates.

Detecting low-abundance/and or low-prevalence microbial features was more challenging in pooled fecal and DNA samples compared to individual samples, highlighting a trade-off in approach when attempting to identify rare taxa in microbiome studies. For example, high-prevalence and high-abundance features were consistently recovered across all our pooling workflows, whereas low-prevalence and low-abundance features were detected more often through the analysis of individual samples (Fig. 4). While increased sequencing depth might improve the detection of very low-abundance taxa, we observed no differences in sequencing depth between individual and pooled samples. Taken together, these considerations suggest that the decision to pool samples should be made thoughtfully, weighing these trade-offs to align with the research objectives.

Farm management practices, including housing, diet, antimicrobial use, differences in animal age, and underlying animal health, may impose considerable heterogeneity on the fecal microbiome of the study population (Gaire et al. 2022), and, in turn, on the pooled microbiome profiles. Limitations of our study include its cross-sectional design, the use of a single farm, and an antibiotic-naive piglet population. Additionally, we compared microbial profiles across pooled workflows obtained from 16S rRNA gene sequencing, which targets genes encoding 16S ribosomal RNA in bacteria (Pace 1997, Caporaso et al. 2011). While this approach is cost-effective, it limits the analysis to bacterial and archaeal taxa and generally provides genus-level taxonomic annotation (and in some cases species-level). A shotgun metagenomic sequencing approach typically provides a more comprehensive microbial profile with higher taxonomic resolution. We hypothesize that the impact of pooling on metagenomic profiles of piglet feces would be similar to what we observed with 16S rRNA gene profiles, but a systematic analysis with shotgun metagenomic data would be needed to test this hypothesis. Furthermore, certain shotgun metagenomic analytical workflows can be affected by pooling. For example, in some situations, pooling individual samples may increase strain-level heterogeneity by introducing a greater diversity of closely related strains from the same species. This increased heterogeneity could impact downstream analyses, particularly those aimed at resolving strain-level differentiation and performing metagenomic assembly (Delgado and Andersson 2022). Additional investigation will be required to better understand the interplay between pooling and strain-level metagenomic analyses, which may be dependent upon the samples being analyzed in any given study.

## Conclusion

The choice between individual and pooled samples for fecal microbiome studies depends on research objectives and available resources. Our findings demonstrate that pooling raw feces or extracted DNA offers a cost-effective approach that can be used to estimate microbial diversity, abundance, and composition at the litter level in preweaned piglets. However, researchers should be aware that pooling may not capture low-abundance or low-prevalence taxa, which are more reliably detected through the analysis of individual samples. Additionally, composite pen-floor samples are unsuitable for characterizing microbiome profiles at either the individual or litter level, particularly in young piglets, as they do not accurately represent the microbiome of individual piglets or pooled samples. While pooling is a viable approach for group-level microbiome studies, understanding these trade-offs is essential for researchers to make informed decisions about when and how to employ pooling in fecal microbiome studies. To maximize the utility of pooling in population-level microbiome studies, future research is needed to evaluate how effectively pooled samples capture microbiome dynamics across different livestock species and production settings. Ideally, such studies should investigate factors such as the optimal number of samples within pools, the ability of pooling methods to represent overall microbial composition, the detection of low-prevalence and low-abundance taxa, and the accuracy of pooled samples in reflecting temporal changes or responses to interventions. These efforts will help refine pooling methods, ensuring their applicability to diverse production systems, environmental conditions, and management practices in livestock microbiome studies.

## Supplementary Material

fiaf058_Supplemental_File

## Data Availability

The raw sequence data generated from 16S rRNA gene sequencing in this study are available in the NCBI repository under BioProject PRJNA1162863.
