# Supplementary material for: The impact of pooling on the observed microbiome profile of preweaned piglet feces
Source: FEMS Microbiol Ecol. 2025 Jun 6;101(6):fiaf058. doi: 10.1093/femsec/fiaf058 (PMC12166543; doi:10.1093/femsec/fiaf058)
Supplement: fiaf058_Supplemental_File [file fiaf058_supplemental_file.docx]

**Supplementary Files**

**Supplementary Figures and Tables**


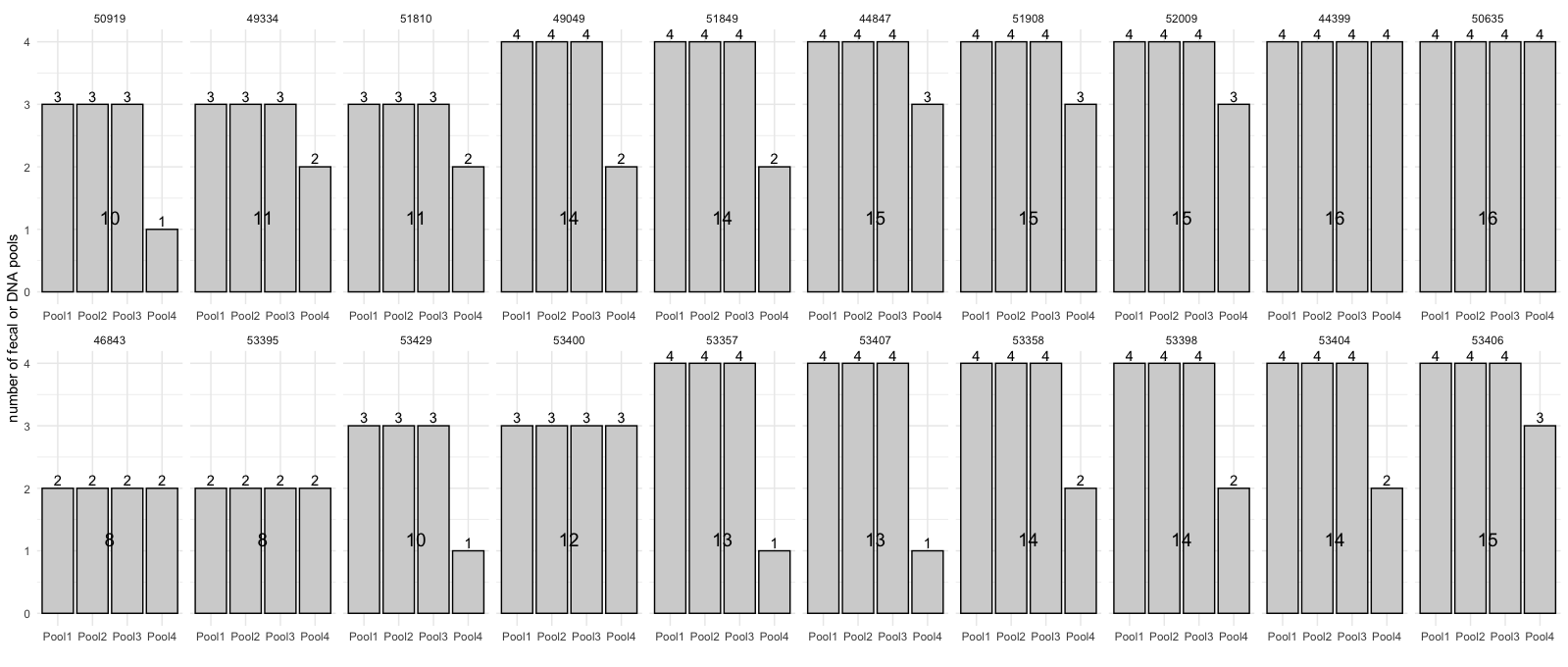


**Fig. S1.** Number of fecal or DNA pools formed from individual samples for each litter/Sow ID (N=20). Each subplot corresponds to a different litter ID/Sow ID. The numbers on top of each bar represent the number of individual piglet samples used for pooling. The numbers in the middle of each plot indicate the total litter size for the given litter ID/Sow ID.

**Table S1**. Composition of the lactation diet for sows.

| **Ingredient** | **Inclusion (%)** |
| --- | --- |
| Corn | 56.3 |
| Soybean Meal (SBM) | 23.1 |
| Corn Distillers Dried Grains with Solubles (DDGS) | 15.0 |
| Limestone | 1.5 |
| Monocalcium Phosphate (21%) | 1.0 |
| Fat (Animal-Vegetable Blend) | 1.0 |
| Potassium Chloride | 0.6 |
| Salt | 0.5 |
| L-Lysine HCl | 0.4 |
| Vitamin and Trace Mineral Premix (VTM) | 0.2 |
| Choline Chloride | 0.1 |
| L-Threonine | 0.1 |
| Mycotoxin Binder | 0.1 |
| L-Tryptophan | 0.0 |
| DL-Methionine | 0.0 |

**Table S2**. Composition of the creep feed.

| **Ingredient** | **Inclusion (%)** |
| --- | --- |
| Corn Ground 6.8% | 27.67 |
| Whey Edible | 22.05 |
| Feeding Oatmeal Bulk | 20.0 |
| SBM 46% | 18.0 |
| Fish Meal/ IPC 790 | 6.50 |
| Animal Fat/Choice White | 1.62 |
| HP 300 | 1.25 |
| Organic and inorganic acids* | 0.50 |
| L-Lysine 99% | 0.49 |
| Zinc Oxide 72% | 0.34 |
| Dl-Methionine 99% | 0.26 |
| Salt/Mix & Fines | 0.25 |
| THR Pro 80% | 0.21 |
| Calcium Carb 39% | 0.20 |
| Maxi-Bond | 0.13 |
| L-Valine 98.5% | 0.11 |
| Choline Chlor 60% | 0.08 |
| (I) TM Swine TM (No Se) | 0.08 |
| L-Tryptophan 98% | 0.08 |
| (I)Sow Vitamin PMX | 0.07 |
| Copper Sulfate 25.2% | 0.05 |
| Phytase 7,000 HS | 0.03 |
| Ethoxyquin 66 2/3% | 0.01 |
| SEL-Plex 3000 (Org Se) | 0.01 |
| Vit E 50% | 0.0 |

***Kemin Industries, Inc.**


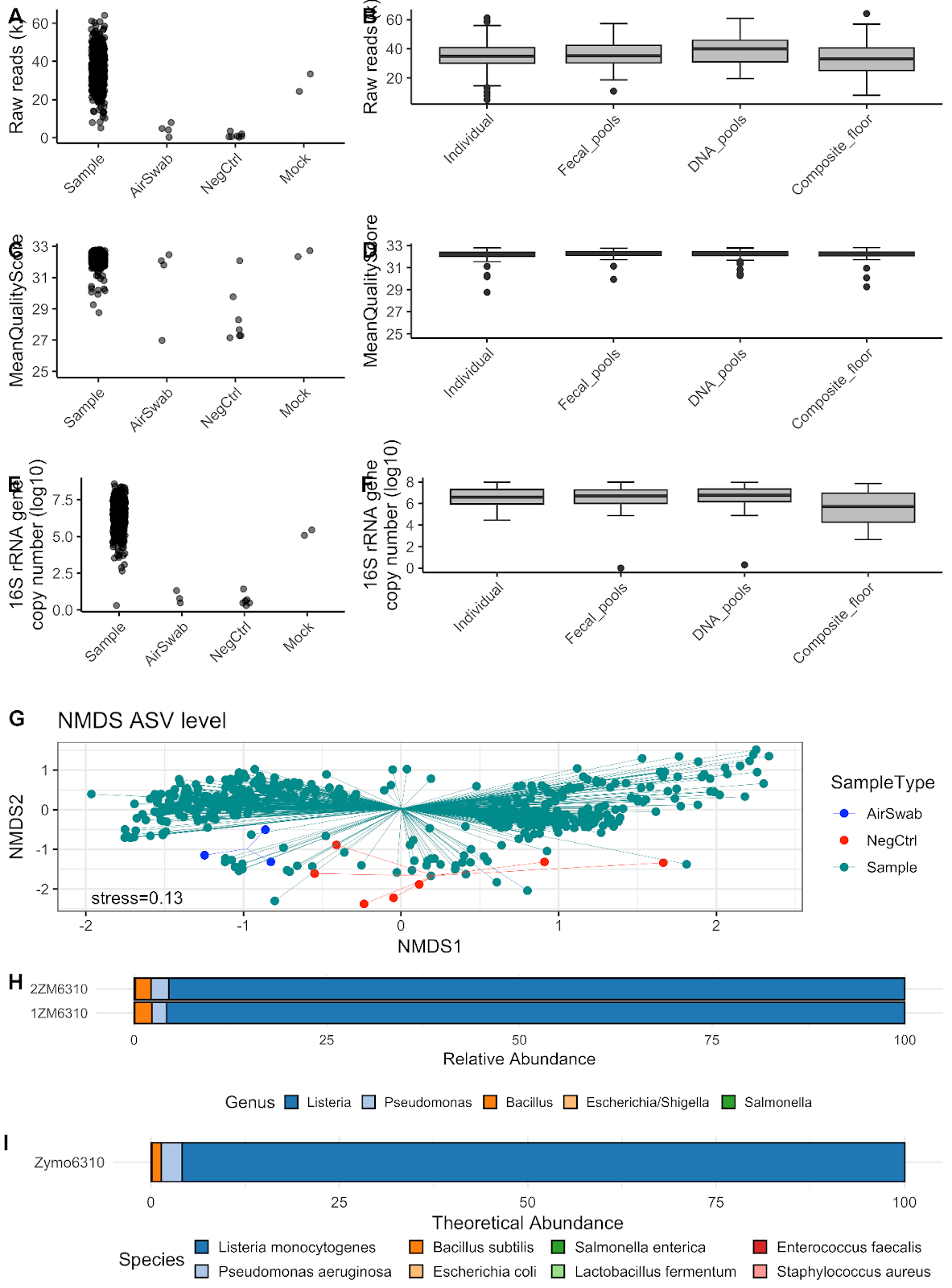


**Fig. S2.** Sequencing metrics, 16S rRNA gene copy number, and overall microbial composition across sample types. (A) Number of raw reads for samples, sampling controls (i.e., air blanks, extraction blanks), and mock communities. (B) Number of raw reads for individual fecal swabs, fecal pools, DNA pools, and composite pen-floor swabs. (C) Mean quality scores of raw reads for samples, sampling controls (i.e., air blanks, extraction blanks), and mock communities. (D) Mean quality scores of raw reads for individual fecal swabs, fecal pools, DNA pools, and composite pen-floor swabs. (E) 16S rRNA gene copy number (log10) for samples, sampling controls (i.e., air blanks, extraction blanks), and mock communities. (F) 16S rRNA gene copy number (log10) for individual fecal swabs, fecal pools, DNA pools, and composite pen-floor swabs. (G) Non-metric multidimensional scaling (NMDS) plot based on ASV-level Bray-Curtis dissimilarities, showing the clustering of samples and sampling controls (PERMANOVA *P*=0.01). (H) Observed relative abundance of taxa in the ZymoBIOMICS Microbial Community Standard II (Log Distribution, catalog # D6310). (I) Theoretical relative abundance of taxa in the ZymoBIOMICS Microbial Community Standard II (Log Distribution, catalog # D6310). Box plots display the median, interquartile range, and outliers. Individual data points are shown as scatter points.


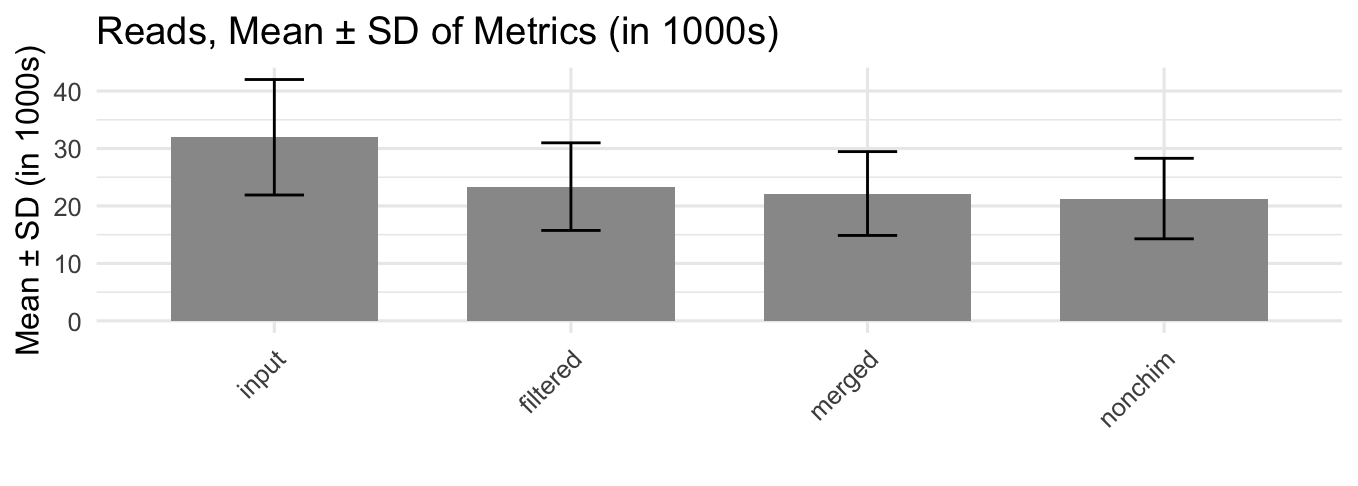


**Fig. S3.** Number of raw reads retained at each processing step of the DADA2 bioinformatics pipeline. Each bar represents the average number of reads retained across all samples, with error bars indicating the standard deviation. Input - Reads obtained after primer removal, Filtered - reads retained after quality filtering (removal of low-quality and ambiguous sequences), Merged - successfully merged paired forward and reverse reads, Nonchim - non-chimeric reads retained after chimera removal.

**Table S3.** Summary of reads retained at each step of the DADA2 bioinformatics pipeline by pooled workflow and sampling controls (Mean ± SD, in 1000s).

| Pooled workflow/SampleType | N | Input | Filtered | Merged | Non-Chimeric |
| --- | --- | --- | --- | --- | --- |
| Individual | 258 | 32.23 ± 8.67 | 23.47 ± 6.59 | 22.36 ± 6.31 | 21.31 ± 6.01 |
| Fecal_pools | 80 | 32.96 ± 8 | 24.22 ± 6.17 | 22.97 ± 5.91 | 22.1 ± 5.74 |
| DNA_pools | 80 | 35.21 ± 9.54 | 25.85 ± 7.37 | 24.55 ± 7.09 | 23.61 ± 6.82 |
| Composite_floor | 60 | 30.58 ± 10.74 | 22.45 ± 8.19 | 20.88 ± 7.77 | 20.66 ± 7.63 |
| Sampling Blank (i.e. AirBlank) | 4 | 3.9 ± 2.9 | 2.84 ± 2.29 | 2.61 ± 2.24 | 2.61 ± 2.24 |
| ExtractionBlank | 7 | 1.09 ± 1.05 | 0.56 ± 0.82 | 0.48 ± 0.74 | 0.48 ± 0.74 |
| Mock | 2 | 23.31, 30.60 | 16.46, 24.10 | 16.27, 23.92 | 16.12, 23.51 |


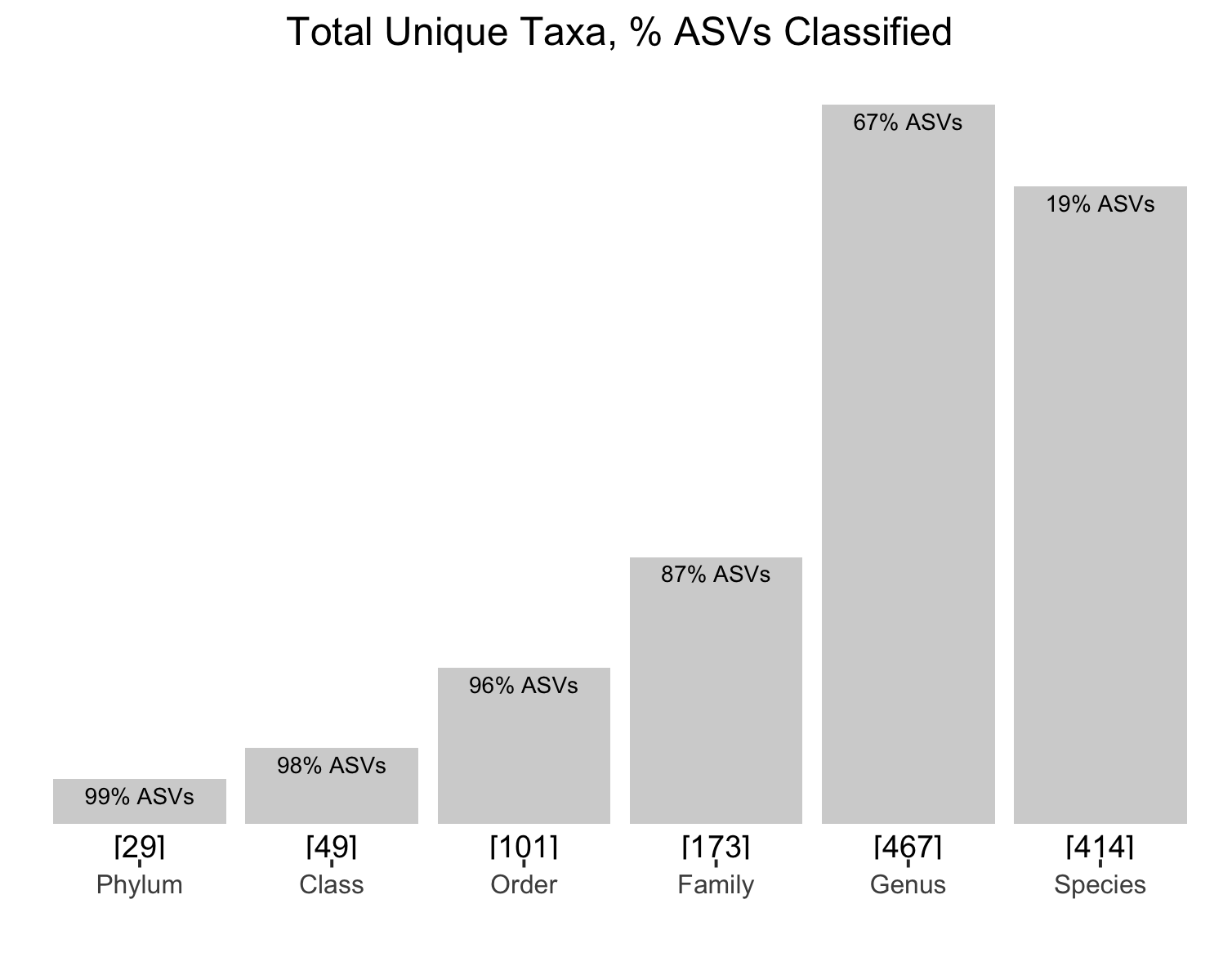


**Fig. S4.** Percentage of ASVs assigned to each taxonomic level based on classification using the SILVA 138.2 database in the decontaminated data. The numbers below each bar indicate the total number of unique taxa identified at each level: Phylum, Class, Order, Family, Genus, and Species. The percentages above each bar represent the percentage of ASVs classified to each respective level.

**
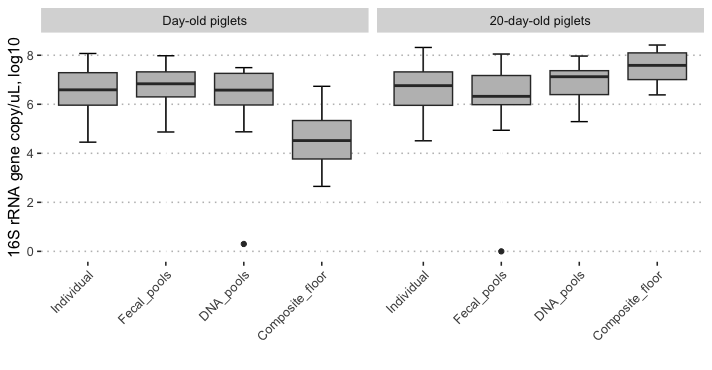
Fig. S5.** Comparison of 16S rRNA gene copy number (log10) across pooled workflows. Box plots display the distribution of 16S rRNA gene copy number for individual samples, fecal pools, DNA pools, and composite floor samples, separated by piglet age (day-old and 20-day-old). Boxes represent the median and interquartile range.

**
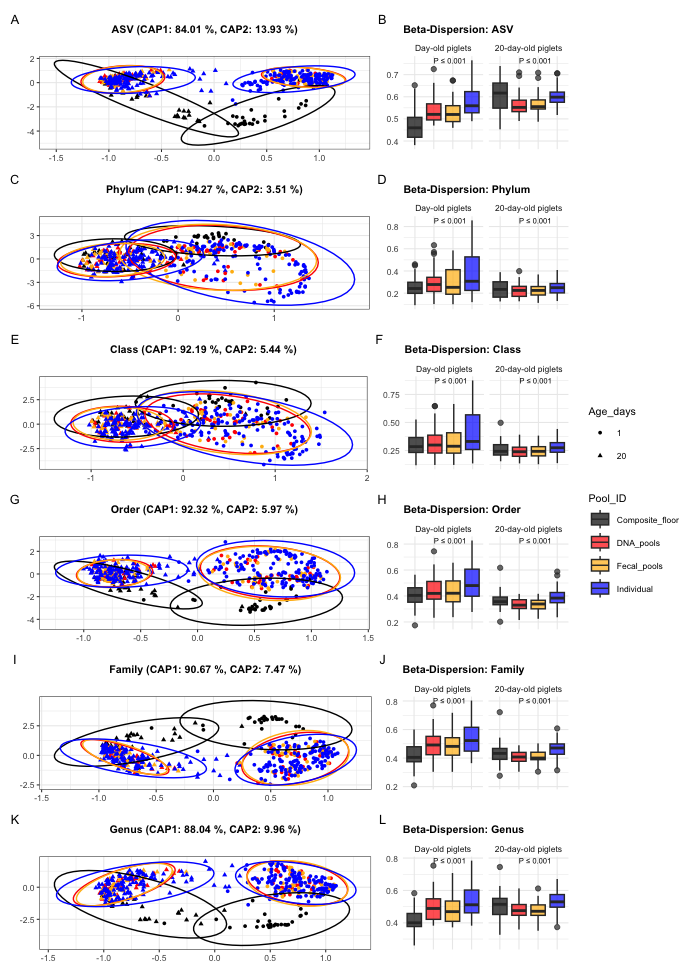
**

**Fig. S6.** Redundancy analysis (db-RDA) based on Bray-Curtis dissimilarities, illustrating the microbial composition of pooled workflows at various taxonomic levels for day-old (filled circles) and 20-day-old (triangles) piglets. Panels A-L show microbial composition for individual samples (blue), fecal pools (yellow), DNA pools (red), and composite floor samples (black). Each point represents a sample, and ellipses represent the 95% confidence interval around the group centroid. Adjacent boxplots display beta-dispersion (distance to group centroid) stratified by piglet age and workflow. Boxplots show the median, interquartile range, and whiskers extending to 1.5 times the interquartile range, with outliers plotted individually.

**
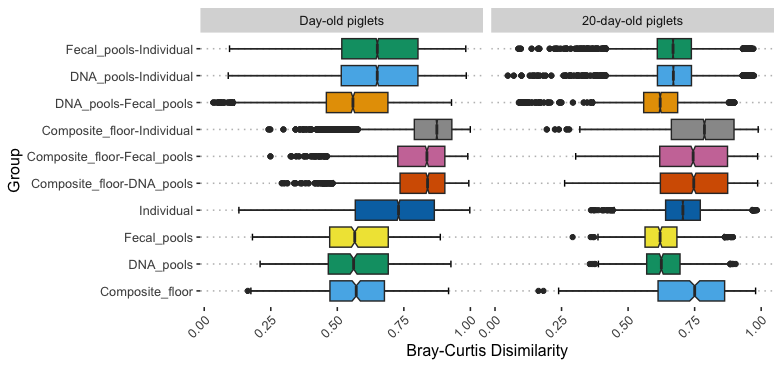
**

**Fig. S7.** Pairwise Bray-Curtis dissimilarities at the ASV level, comparing microbial community composition among different pooling methods for day-old and 20-day-old piglets. Box plots display the within and between Bray-Curtis dissimilarities between pooled workflow. Box plots show the median, interquartile range, and outliers.


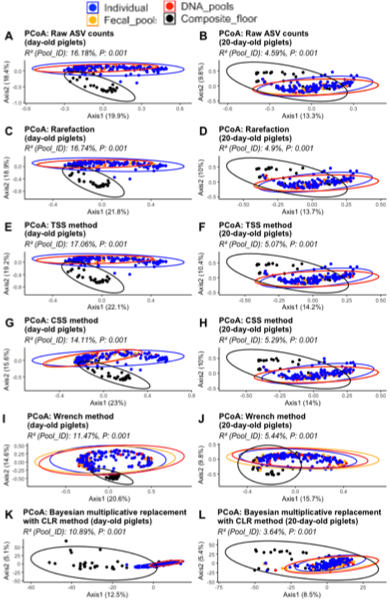


**Fig. S8.** Principal Coordinate Analysis based on Bray-Curtis dissimilarities or Aitchison distance of ASV-level data, comparing normalization methods and age groups in piglets. PCoA plots are shown for day-old (left column) and 20-day-old (right column) piglets using the following normalization methods: (A, B) Raw ASV counts (i.e., not normalized or transformed); (C, D) Rarefaction (even depth); (E, F) Total Sum Scaling; (G, H) Cumulative Sum Scaling; (I, J) Wrench normalization; and (K, L) Bayesian multiplicative replacement ("BL") with centered log-ratio transformation. Ellipses represent 95% confidence intervals for each sample type: composite floor (black), fecal pools (orange), DNA pools (red), and individual samples (blue). *R²* and *P*-values indicate the variation partitioned to the pooled workflow and statistical significance, respectively*.* The percent variation explained by Axis 1 and Axis 2 is shown in parentheses.


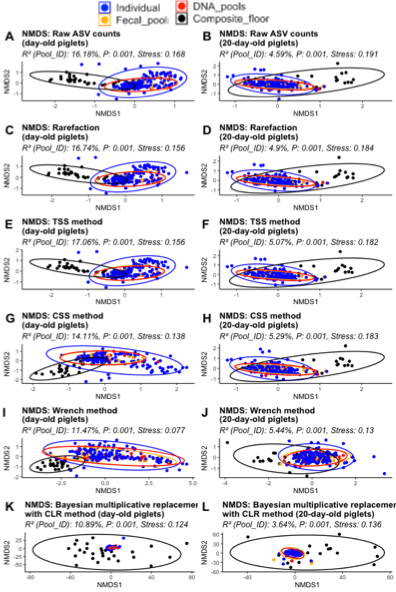


**Fig. S9.** Non-metric multidimensional scaling ordination based on Bray-Curtis dissimilarities or Aitchison distance of ASV-level data, comparing normalization methods and age groups in piglets. NMDS plots are shown for day-old (left column) and 20-day-old (right column) piglets using the following normalization methods: (A, B) Raw ASV counts (i.e., not normalized or transformed); (C, D) Rarefaction (even depth); (E, F) Total Sum Scaling (TSS); (G, H) Cumulative Sum Scaling (CSS); (I, J) Wrench normalization; and (K, L) Bayesian multiplicative replacement ("BL") with centered log-ratio transformation for Aitchison distance calculation. Ellipses represent 95% confidence intervals for each sample type. Stress values indicate ordination quality. *R²* and *P*-values indicate the variation partitioned to the pooled workflow and statistical significance, respectively.

**
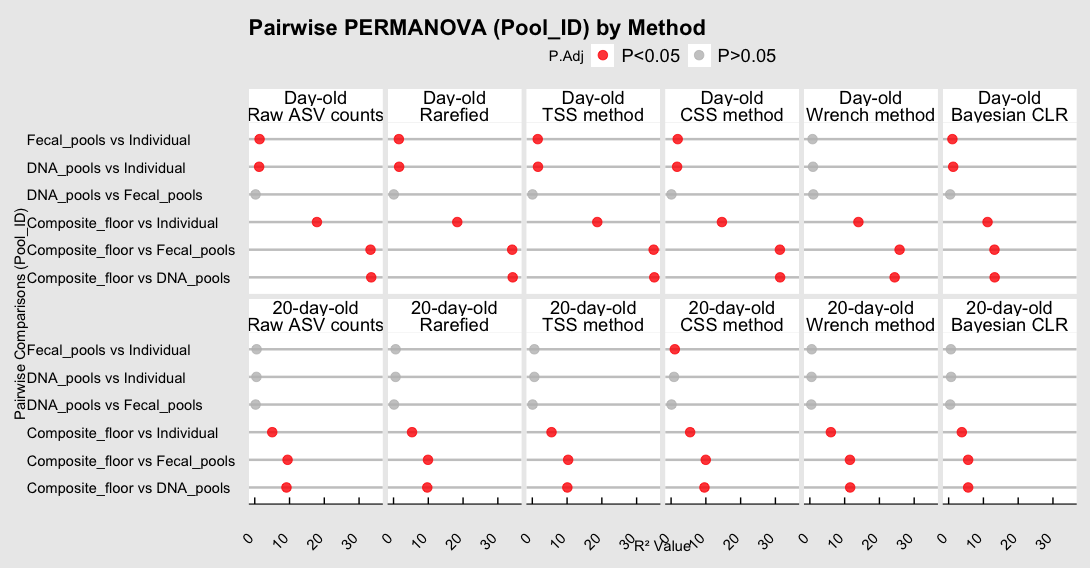
**

**Fig. S10.** Pairwise PERMANOVA results for pooled workflows across normalization/ transformation methods and age groups in piglets. The x-axis displays *R*² values (proportion of variation partitioned to the workflow), and the y-axis shows all possible pairwise comparisons between pooled workflows (e.g., fecal pools vs. individual, DNA pools vs. individual). Each panel represents a normalization method and age group: raw ASV counts, rarefaction, Total Sum Scaling (TSS), Cumulative Sum Scaling (CSS), Wrench normalization, and Bayesian multiplicative replacement ("BL") with centered log-ratio transformation (CLR) for day-old (top) and 20-day-old (bottom) piglet samples. Red points indicate statistically significant pairwise comparisons (Benjamini-Hochberg adjusted *P*-value < 0.05), while gray points represent non-significant comparisons (adjusted *P-*value ≥ 0.05)*.*

**
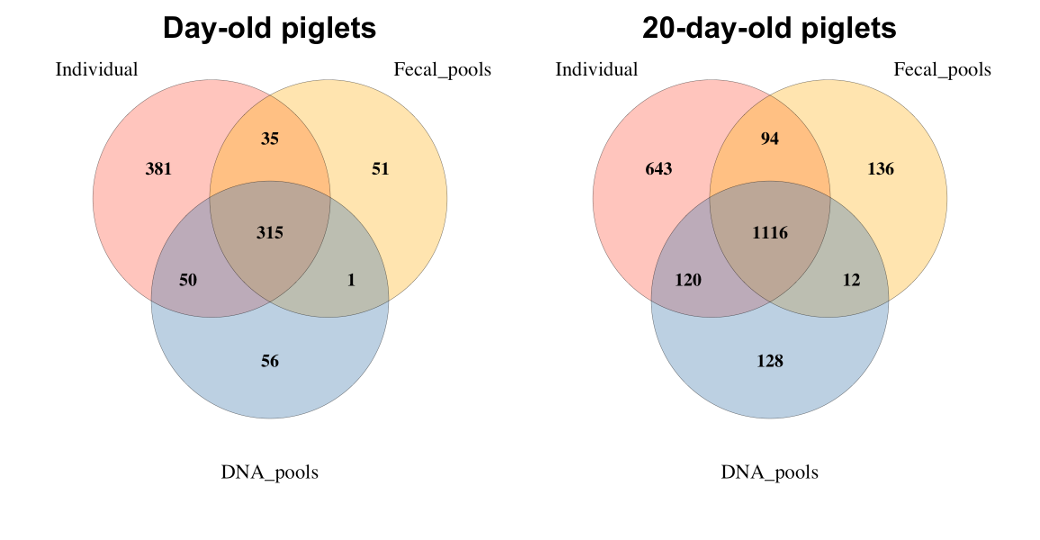
**

**Fig. S11.** Venn diagrams comparing the ASVs among individual samples, fecal pools, and DNA pools, excluding composite floor samples, in day-old piglets and 20-day-old piglets. The numbers within each section of the Venn diagrams represent the count of ASVs unique to each pool type or common between multiple pool types.

**
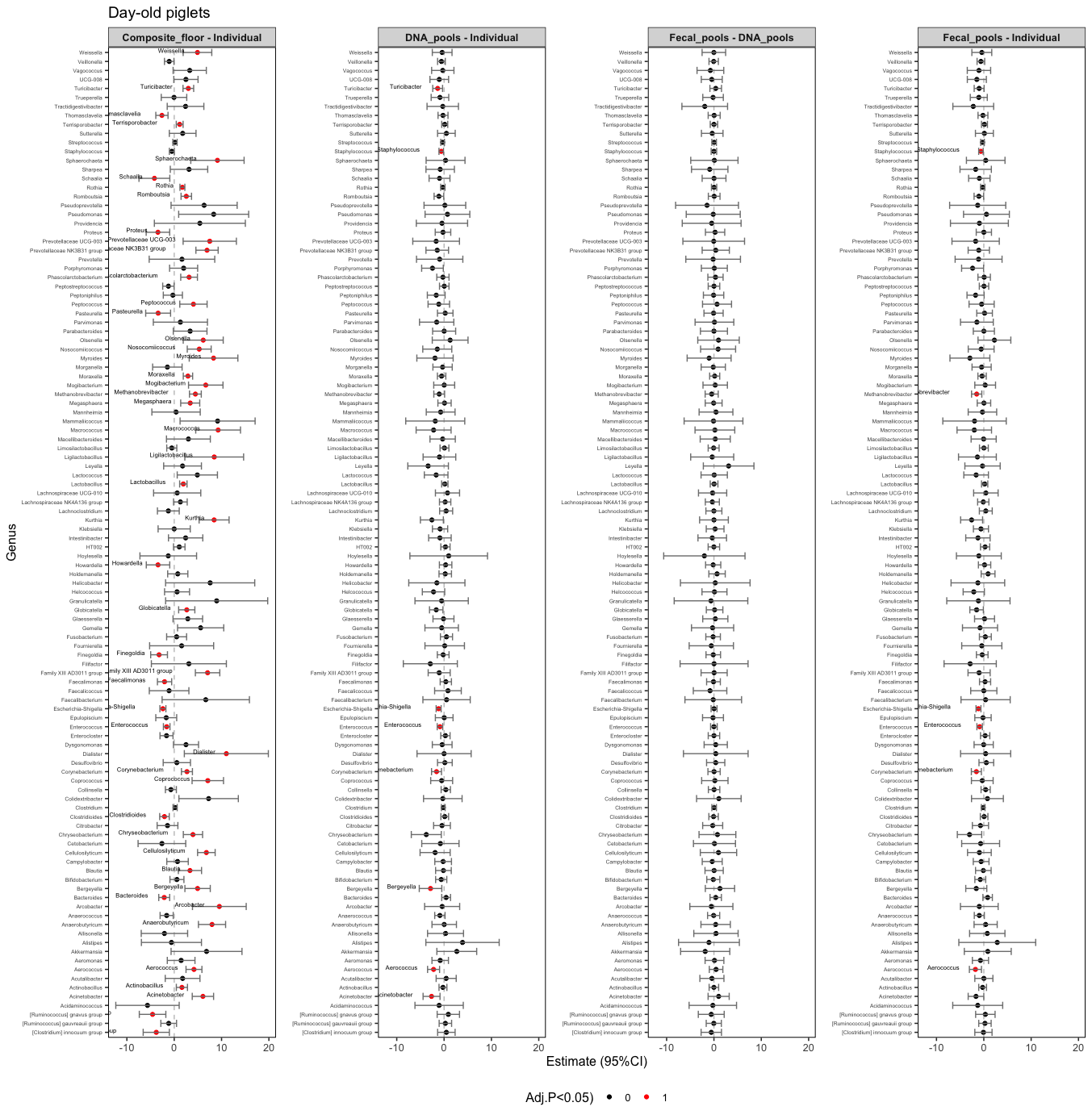
**

**Fig. S12.** Differentially abundant microbial genera between pooled workflows in day-old piglet samples. Each point represents the estimated difference in the abundance of individual microbial genera between pooling workflows, with error bars indicating the 95% confidence intervals (CI).

**
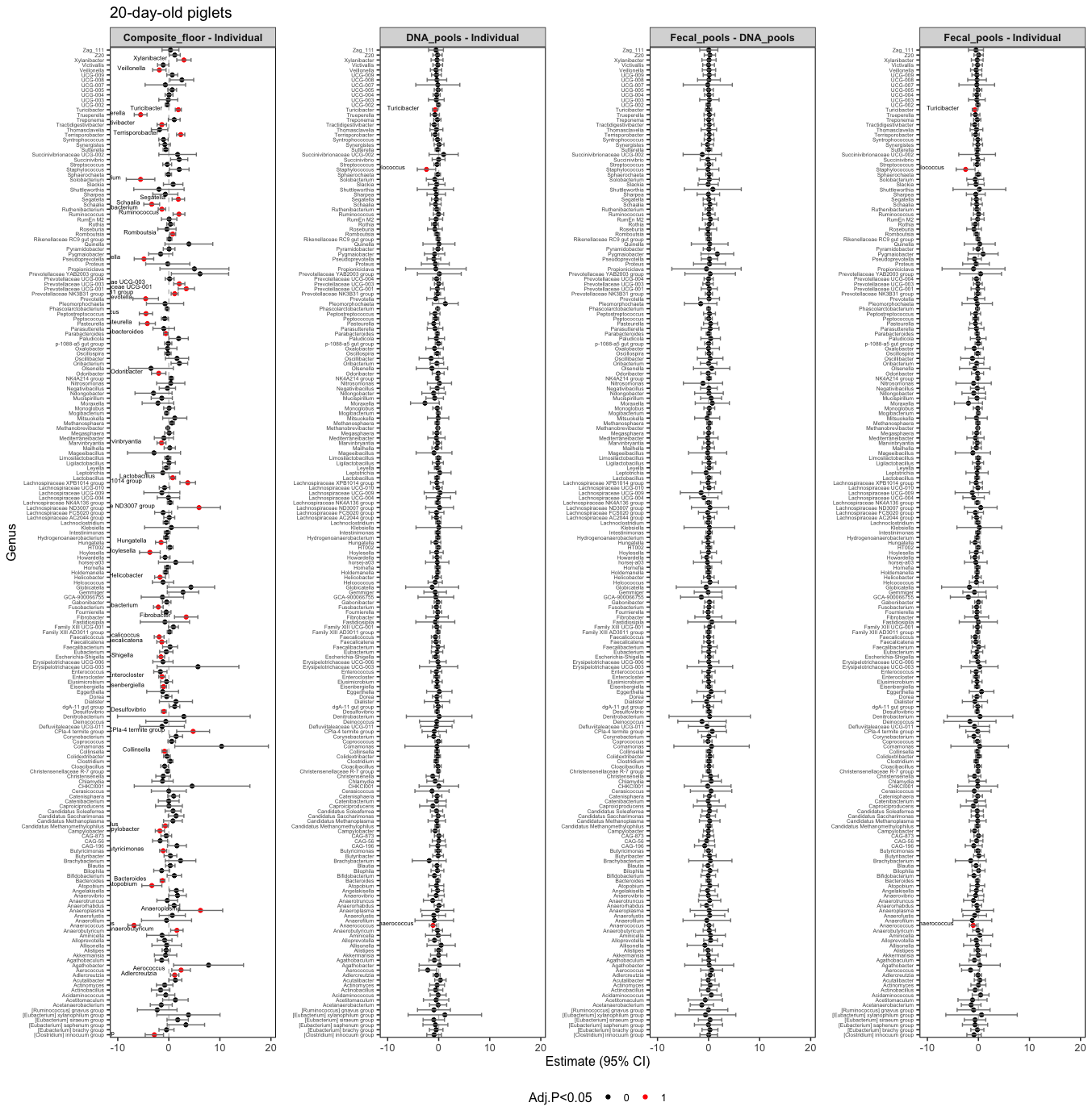
**

**Fig. S13.** Differentially abundant microbial genera between pooled workflows in 20-day-old piglet samples. Each point represents the estimated difference in the abundance of individual microbial genera between pooling workflows, with error bars indicating the 95% confidence intervals (CI).

**Table S4.** Differential abundance of phyla or genera between pooling workflows from the ANCOM-BC2 analysis with CLR-transformed ASV count matrix. Analyses were performed adjusting for covariates (litter size, raw reads) and including litter ID as a random effect, stratified by day-old and 20-day-old piglet samples. The individual samples were used as the reference group. *lfc* represents log-fold change from the model output (i.e. *res_dunn*), where positive and negative *lfc* values indicate significantly higher or lower abundance in the reference group compared to the comparison group and vice-versa. *q* represents the adjusted *P*-values.

| taxon | lfc_Fecal_  pools | lfc_DNA_  pools | lfc_  Composite_  floor | q_Fecal_  pools | q_DNA_  pools | q_Composite_  floor | age_  days | tax_level |
| --- | --- | --- | --- | --- | --- | --- | --- | --- |
| *Methanobacteriota* | -0.07 | -0.11 | 0.85 | 1 | 1 | <0.001 | 1 | Phylum |
| *Actinomycetota* | -0.12 | -0.15 | 1.48 | 1 | 1 | <0.001 | 1 | Phylum |
| *Methanobrevibacter* | -0.43 | -0.48 | 2.49 | 1 | 1 | <0.001 | 1 | Genus |
| *Rothia* | 0.02 | 0.02 | 0.92 | 1 | 1 | <0.001 | 1 | Genus |
| *Kurthia* | -0.07 | -0.03 | 0.67 | 1 | 1 | <0.001 | 1 | Genus |
| *Turicibacter* | 0.03 | 0.03 | 0.48 | 1 | 1 | <0.001 | 1 | Genus |
| *Aerococcus* | -0.14 | -0.06 | 0.7 | 0.98 | 1 | <0.001 | 1 | Genus |
| *Globicatella* | -0.16 | -0.07 | 0.94 | 1 | 1 | <0.001 | 1 | Genus |
| *Enterococcus* | 0.01 | 0.06 | -0.61 | 1 | 1 | <0.001 | 1 | Genus |
| *Lactococcus* | 0.01 | -0.04 | 0.61 | 1 | 1 | 0.006 | 1 | Genus |
| *Macrococcus* | 0 | 0.06 | 0.69 | 1 | 1 | <0.001 | 1 | Genus |
| *Nosocomiicoccus* | -0.08 | -0.11 | 1.1 | 1 | 1 | <0.001 | 1 | Genus |
| *Clostridium* | 0.08 | 0.1 | 0.29 | 1 | 0.83 | 0.001 | 1 | Genus |
| *Coprococcus* | -2.86 | -0.21 | -1.19 | 0.01 | 1 | 0.111 | 1 | Genus |
| *Enterocloster* | 0.16 | 0.09 | -0.9 | 1 | 1 | <0.001 | 1 | Genus |
| *Faecalimonas* | 0.41 | 0.34 | -0.7 | 0.15 | 0.37 | 0.009 | 1 | Genus |
| *Howardella* | 0.22 | 0.31 | -1.36 | 1 | 1 | <0.001 | 1 | Genus |
| *Mogibacterium* | 2.55 | 1.96 | 2.94 | 0.26 | 0.63 | 0.001 | 1 | Genus |
| *Finegoldia* | -0.08 | 0.01 | -1.13 | 1 | 1 | <0.001 | 1 | Genus |
| *Peptostreptococcus* | 0.01 | 0.26 | -0.36 | 1 | 0.08 | 0.023 | 1 | Genus |
| *Romboutsia* | 0.06 | 0.1 | 0.67 | 1 | 1 | <0.001 | 1 | Genus |
| *Terrisporobacter* | 0.11 | 0.1 | 0.45 | 1 | 1 | <0.001 | 1 | Genus |
| *Phascolarctobacterium* | 0.03 | -0.01 | 0.83 | 1 | 1 | 0.001 | 1 | Genus |
| *Allisonella* | -0.15 | 0.07 | -0.84 | 1 | 1 | <0.001 | 1 | Genus |
| *Veillonella* | -0.02 | 0.23 | -0.83 | 1 | 1 | 0.003 | 1 | Genus |
| *Fusobacterium* | -0.02 | 0.12 | -1.47 | 1 | 1 | <0.001 | 1 | Genus |
| *Escherichia-Shigella* | -0.03 | 0.01 | -0.32 | 1 | 1 | 0.001 | 1 | Genus |
| *Glaesserella* | -0.47 | -0.19 | 1.99 | 1 | 1 | 0.001 | 1 | Genus |
| *Pasteurella* | 0.09 | 0.24 | -1 | 1 | 1 | <0.001 | 1 | Genus |
| *Acinetobacter* | 0 | 0 | 5.71 | 1 | 1 | 0.022 | 1 | Genus |
| *Spirochaetota* | 0.05 | 0.11 | 0.34 | 1 | 1 | 0.012 | 20 | Phylum |
| *Candidatus Methanoplasma* | 0.08 | -0.03 | 0.81 | 1 | 1 | 0 | 20 | Genus |
| *Trueperella* | 0.01 | 0.13 | -0.72 | 1 | 1 | 0.006 | 20 | Genus |
| *Brachybacterium* | 1.33 | 0 | 0.64 | 0 | 1 | 0.045 | 20 | Genus |
| *Atopobium* | 0.56 | -0.04 | -5 | 1 | 1 | 0.009 | 20 | Genus |
| *[Clostridium] innocuum group* | -0.76 | -0.73 | -3.44 | 1 | 1 | 0.035 | 20 | Genus |
| *Lachnospiraceae XPB1014 group* | 0.03 | -0.01 | 0.46 | 1 | 1 | 0.011 | 20 | Genus |
| *Anaerococcus* | -0.05 | -0.04 | -0.45 | 1 | 1 | 0.006 | 20 | Genus |
| *Terrisporobacter* | -0.07 | -0.02 | 0.51 | 1 | 1 | 0.002 | 20 | Genus |
| *Treponema* | 0.38 | 0.51 | 1.1 | 1 | 1 | 0.012 | 20 | Genus |
